#  Unique features of HLA-mediated HIV evolution in a Mexican cohort: a comparative study

**DOI:** 10.1186/1742-4690-6-72

**Published:** 2009-08-10

**Authors:** Santiago Avila-Rios, Christopher E Ormsby, Jonathan M Carlson, Humberto Valenzuela-Ponce, Juan Blanco-Heredia, Daniela Garrido-Rodriguez, Claudia Garcia-Morales, David Heckerman, Zabrina L Brumme, Simon Mallal, Mina John, Enrique Espinosa, Gustavo Reyes-Teran

**Affiliations:** 1 Center for Research in Infectious Diseases, National Institute of Respiratory Diseases, Mexico City, Mexico; 2 Faculty of Medicine, National Autonomous University of Mexico, Mexico City, Mexico; 3 eScience Group, Microsoft Research, Redmond, Washington, USA; 4 Ragon Institute of Massachusetts General Hospital, Massachusetts Institute of Technology and Harvard, Boston, Massachusetts, USA; 5 Faculty of Health Sciences, Simon Fraser University, Burnaby, British Columbia, Canada; 6 Center for Clinical Immunology and Biomedical Statistics, Royal Perth Hospital and Murdoch University, Perth, Australia

## Abstract

**Background:**

Mounting evidence indicates that HLA-mediated HIV evolution follows highly stereotypic pathways that result in HLA-associated footprints in HIV at the population level. However, it is not known whether characteristic HLA frequency distributions in different populations have resulted in additional unique footprints.

**Methods:**

The phylogenetic dependency network model was applied to assess HLA-mediated evolution in datasets of HIV *pol *sequences from free plasma viruses and peripheral blood mononuclear cell (PBMC)-integrated proviruses in an immunogenetically unique cohort of Mexican individuals. Our data were compared with data from the IHAC cohort, a large multi-center cohort of individuals from Canada, Australia and the USA.

**Results:**

Forty three different HLA-HIV codon associations representing 30 HLA-HIV codon pairs were observed in the Mexican cohort (q < 0.2). Strikingly, 23 (53%) of these associations differed from those observed in the well-powered IHAC cohort, strongly suggesting the existence of unique characteristics in HLA-mediated HIV evolution in the Mexican cohort. Furthermore, 17 of the 23 novel associations involved HLA alleles whose frequencies were not significantly different from those in IHAC, suggesting that their detection was not due to increased statistical power but to differences in patterns of epitope targeting. Interestingly, the consensus differed in four positions between the two cohorts and three of these positions could be explained by HLA-associated selection. Additionally, different HLA-HIV codon associations were seen when comparing HLA-mediated selection in plasma viruses and PBMC archived proviruses at the population level, with a significantly lower number of associations in the proviral dataset.

**Conclusion:**

Our data support universal HLA-mediated HIV evolution at the population level, resulting in detectable HLA-associated footprints in the circulating virus. However, it also strongly suggests that unique genetic backgrounds in different HIV-infected populations may influence HIV evolution in a particular direction as particular HLA-HIV codon associations are determined by specific HLA frequency distributions. Our analysis also suggests a dynamic HLA-associated evolution in HIV with fewer HLA-HIV codon associations observed in the proviral compartment, which is likely enriched in early archived HIV sequences, compared to the plasma virus compartment. These results highlight the importance of comparative HIV evolutionary studies in immunologically different populations worldwide.

## Background

The cytotoxic CD8+ T lymphocyte (CTL) response has been identified as an important selective pressure driving HIV evolution within an infected host [1-5]. Strong lines of evidence support the importance of the CTL response in HIV control, including the temporal correlation between the appearance of HIV-specific CTLs in vivo and the decline of viremia in the early stages of HIV infection [6], as well as the lack of control of virus levels after experimental depletion of CD8+ cells in rhesus macaques prior to simian immunodeficiency virus (SIV) infection [7]. CTLs recognize and destroy infected cells through the binding of their T cell receptor (TCR) to viral peptides (epitopes) presented on the surface of infected cells by highly polymorphic molecules encoded by class I human leukocyte antigen (HLA) genes. Each HLA allele encodes a unique HLA molecule capable of presenting a broad range of possible epitopes derived from various areas of the HIV proteome. CTL recognition of these peptide-HLA complexes may be associated with different functional outcomes in the infection [8-10]. Importantly, as a result of CTL-mediated selective pressure, immune escape mutations are selected that hinder viral peptide binding to HLA molecules, prevent peptide processing before their presentation or lower TCR affinity of specific CTL clones to peptide-HLA complexes [4,11-13]. Therefore, both the processes of antigen presentation to CTLs and CTL escape are HLA-restricted [14].

Depending on their costs to viral fitness, some CTL escape mutations can be transmitted and maintained in a new host [15-18], even without the presence of the originally selective HLA allele [11,19-21]. Additionally, there is evidence supporting the notion that some immune escape mutations can accumulate in a large number of individuals and become fixed in the circulating virus consensus sequence, driving HIV evolutionary changes at the population level [9,19,22-24]. As a result, specific HLA epitopes could become extinct in the viral population, allowing HIV adaptation to HLA-associated immune control in a certain region [22,23]. The relative impact of different factors that could influence the persistence of escape mutations in a large number of individuals remains incompletely understood [22,25]. The variety of these factors–such as the extent of reversion of immune escape mutations in the absence of the selecting HLA allele [15,16,18], selection of compensatory mutations that restore viral fitness [26], founder effects [9], conflicting evolutionary forces on clustered epitopes [27], development of novel CTL responses to escape variants [28,29], inter-clade differences in the circulating viruses [14,30], immunodominance hierarchies of CTL responses [25,31,32], and HLA allele frequency distributions in different populations [22]–highlight the complexity of viral adaptation to the immune response at the population level [9,25].

In spite of this complexity, mounting evidence indicating that a large number of CTL escape mutations are reproducibly selected in the context of specific HLA restrictions has led to the hallmark observation that HIV evolution follows generally predictable mutational patterns in response to specific HLA-restricted immune responses (reviewed in [10]). This "HLA footprint effect" on HIV has been shown at the population level through correlative associations between the presence (or absence) of polymorphisms at specific positions of the viral sequence and the expression of specific HLA alleles [24,25,33-35]. Detection of HLA-HIV polymorphism associations is potentially limited by important confounding effects, namely HIV phylogeny, HIV codon covariation, and linkage disequilibrium of HLA alleles [10,14]. Several studies have accounted for some of these confounding effects explicitly [24,30,34]; more recently, a comprehensive evolutionary model considering all these confounding sources was proposed [14]. This phylogenetic dependency network model was shown to be able to reconstruct previously defined escape and compensatory mutation pathways and agrees with emerging data on patterns of epitope targeting. The existence of this kind of comprehensive models represents an opportunity to systematically study HIV evolution in immunogenetically different populations and assess the importance of different HLA backgrounds in HIV evolution at the population level. Due to the extensive polymorphism of HLA genes, allelic and haplotypic frequency distributions in distinct infected populations vary widely [9]. Given the highly consistent effect of HLA-restricted selection on HIV evolution and the distinct HLA allele distributions in differing populations, it is likely that specific HLA-HIV polymorphism associations will be preferentially observed in different populations, determining unique characteristics of HIV evolution in different human groups [9,36]. To explore this possibility, HLA-mediated HIV evolution at the population level was studied in a cohort of clade B-infected individuals from Central/Southern Mexico, and compared to previously reported studies in a large multicenter cohort of predominantly clade B-infected individuals from British Columbia, Canada; Western Australia; and the USA (the International HIV Adaptation Collaborative [IHAC] cohort) (Brumme ZL, John M, et al, PLoS ONE 2009, in press) [14,25,34,37]. In order to determine to what extent HLA imprinting on HIV is a general phenomenon, it is informative to study HIV evolution in an immunogenetically unique population that possibly reflects a different selective pressure to that observed in other studied populations. The Mexican population is known to have a unique immunogenetic background characterized by the admixture of mainly Amerindian and Caucasian HLA haplotypes [38,39]. To our knowledge, Latin American cohorts have not been the primary subject of HIV evolutionary studies. Our data suggest that the unique HLA frequency distribution in a previously uncharacterized, immunogenetically unique HIV-infected population is imprinting HIV evolution in a unique way. This fact underlines the importance of systematically expanding our understanding of CTL escape and HIV evolution in immunogenetically distinct populations. This knowledge has important implications for the design of CTL-based vaccines and treatment strategies.

## Methods

### Study population

Peripheral blood samples were prospectively obtained from 303 chronically-infected, HIV positive, antiretroviral treatment-naïve individuals from Central/Southern Mexico. Participating individuals were recruited with written informed consent at different health centers in Mexico City and from the states of Puebla, Jalisco, Oaxaca, Guerrero, the State of Mexico and Chiapas. Blood samples were shipped to and processed at the Center for Research in Infectious Diseases of the National Institute of Respiratory Diseases in Mexico City. All ethical issues related to this project were evaluated and approved by the Institutional Bioethics and Science Committee. For each patient, plasma aliquots and peripheral blood mononuclear cells (PBMCs) were obtained and cryopreserved.

HLA frequency and HLA-mediated HIV evolution data obtained from the Mexican cohort were compared with that obtained from a previously described cohort of 1,045 HIV-positive, predominantly Caucasian individuals from British Columbia, Canada (HOMER cohort) [34] and the large multicenter International HIV Adaptation Combined (IHAC) cohort, including 1,845 predominantly Caucasian individuals from British Columbia, Canada; Western Australia and the USA [37] (Brumme ZL, John M, et al, PLoS ONE 2009, in press).

### HLA typing

Genomic DNA was extracted from at least 6 million PBMCs using QIAmp DNA Blood Mini Kit (QIAGEN, Valencia CA), according to the manufacturer's specifications. Class I HLA A, B and C genes were typed at low/medium resolution for each participating individual by sequence-specific primer polymerase chain reaction (SSP-PCR) using ABC SSP UniTray Kit (Invitrogen, Brown Deer, WI) according to the manufacturer's specifications. Briefly, genomic DNA from each participating individual at 75–125 ng/μL was used as template for 95 PCRs with different sequence-specific primers designed to detect relevant polymorphisms for typing. Reaction products were run on a 2.0% agarose gel (Promega, Madison, WI). Amplification patterns were analyzed with UniMatch v3.2 software using up-to-date data bases to determine HLA groups. All the reactions included an internal amplification control to be validated and each test included a reagent control to detect contamination.

### HLA frequency analyses and comparisons

HLA allelic and population frequencies for the Mexican cohort were obtained with the HLA Frequency Analysis tool of the Los Alamos HIV Database . HLA haplotype frequencies were obtained with the Arlequin v3.11 software. Due to the fact that the cohort was composed of non-related individuals with unknown family genetic backgrounds, a gametic phase estimation was carried out for each individual using a pseudo-Bayesian algorithm designed to reconstruct the gametic phase of multi-loci genotypes, included in the Arlequin v3.11 software (Excoffier-Laval-Balding, ELB) [40]. Frequency analysis between the cohort reported here and the Canadian HOMER cohort, the multicenter IHAC cohort and a cohort of HIV-negative individuals from Central/Northern Mexico [38], was carried out by chi squared test, with post hoc two by two significance determined by Fisher's exact test, corrected for multiple comparisons by q values [41]. Significant values were considered to be q < 0.05. These analyses were carried out with R statistical environment v2.8.1, using the package qvalue v1.1.

### HIV *pol *genotyping from free plasma virus

Viral RNA from free plasma virus was purified from 1 mL of plasma using QIAmp Viral RNA Mini Kit (QIAGEN, Valencia, CA) according to the manufacturer's specifications. A fragment of the viral *pol *gene including the whole protease (PR) and 335 codons of the reverse transcriptase (RT) was bulk sequenced from plasma viral RNA for each participating individual. Sequences were obtained with a 3100-Avant Genetic Analyzer (Applied Biosystems, Foster City, CA), using ViroSeq HIV-1 Genotyping System (Celera Diagnostics, Alameda, CA) according to the manufacturer's specifications. Briefly, 1.3 Kbp fragments of the *pol *gene were amplified by RT-PCR from plasma viral RNA. PCR products were purified with ultra filtration columns and quantified in 1.5% agarose gels (Promega, Madison, WI). For each patient, sequencing PCRs were carried out with 7 different primers to assure that the whole genomic region was covered with at least two sequences. Sequences were assembled, aligned to the HXB2 consensus, and manually edited using the ViroSeq v2.7 software provided by the manufacturer.

### HIV *pol *genotyping from PBMC proviral DNA

Genomic DNA was purified as described above. A fragment of approximately 1.5 Kbp covering the whole PR and the first 335 codons of RT was amplified by nested PCR with Platinum Taq DNA Polymerase (Invitrogen, Carlsbad, CA), and primers PR 5' OUTER 5'-CCCTAGGAAAAAGGGCTGTTG-3'/RT 3' OUTER 5'-GTTTTCAGATTTTTAAATGGCTCTTG-3', for the first round of amplification, and PR 5' INNER 5'-TGAAAGATTGTACTGAGAGACAGG-3'/RT 3' INNER 5'-GGCTCTTGATAAATTTGATATGTCC-3' for the second round of amplification. PCR conditions were 1 cycle of 94°C, for 3 min, followed by 35 cycles of 94°C for 30 s, 60°C for 30 s and 72°C for 2 min and a cycle of 72°C for 5 min, with final concentrations of 2 mM Mg^++^, 0.2 mM dNTPs, 0.4 mM of each primer and 20 ng/μL genomic DNA for both amplification rounds (transferring 10% of the volume of first round PCR product to the second round). In all cases, contamination controls were included. PCR products were purified by QIAquick PCR Purification Kit (QIAGEN, Valencia, CA) according to the manufacturer's specifications, and quantified in 2.0% agarose gels (Promega, Madison, WI). Seven sequencing PCRs were carried out for each patient using seven primer mixes included in the ViroSeq HIV-1 Genotyping System Kit (Celera Diagnostics, Alameda, CA), in order to cover the whole analyzed region with at least two sequences. Bulk proviral *pol *sequences were obtained with a 3100-Avant Genetic Analyzer (Applied Biosystems, Foster City, CA). Sequences were assembled, aligned to the HXB2 consensus, and edited manually using the ViroSeq v2.7 software.

### Evolutionary analyses

The phylogenetic dependency network (PDN) model by Carlson, et al [14], was applied to infer patterns of CTL escape and codon covariation in the plasma and proviral sequence datasets, using the PhyloDv program . The PDN model was designed to simultaneously account for HIV codon covariation, linkage disequilibrium among HLA alleles and the confounding effects of HIV phylogeny when attempting to identify HLA-associated polymorphisms in HIV [14]. Briefly, the PDN model is a multivariate model that represents the probabilistic dependencies among a set of target attributes (in this case the presence or absence of amino acids at all codons in an HIV protein) and a set of predictor attributes (in this case the presence or absence of amino acids at all codons other than that for the target attribute in the HIV sequence, as well as the presence or absence of all possible HLA alleles) while correcting for the phylogenetic structure of the viral sequences. A dependency network graphically depicts which HLA and codon attributes predict each target codon attribute, associating a probability distribution for each target codon attribute, conditioned on various HLA and codon attributes. Importantly, each local probability distribution is corrected for the phylogenetic structure of the HIV sequences. To determine the significance of a particular predictor-target pair, the likelihood of a null model that reflects the assertion that the target variable is not under selection pressure from the predictor attribute is compared to an alternative model that reflects the assertion that the target variable is under selection pressure from that predictor attribute. Multiple predictors are added to the model in an iterative fashion using forward selection, in which the most significantly associated attribute is iteratively added to the model until no attribute achieves p < 0.05. The use of a multivariate model minimizes spurious associations caused by the presence of linkage disequilibrium among HLA alleles and HIV codon covariation. For each added predictor attribute, the most significant leaf distribution is recorded (escape, reversion, attraction, or repulsion, see below). The statistical significance of a predictor with respect to a target attribute is computed using false discovery rates (FDRs), which are computed using a likelihood-ratio test in which both the null and the alternative models are conditioned on all significant predictors that were identified in previous iterations of forward selection. For each p-value, we report the corresponding q-value, which is the minimum FDR among rejection regions that include that p-value, as computed using the method of Storey and Tibshirani with the π_0 _parameter conservatively set to one [41]. Attributes were excluded as possible predictors when the corresponding predictor-target pair had a 2 × 2 contingency table in which any cell of the table had an observed or expected value of three or less.

The precise rules governing the transitions of the target attribute, conditioned on the predictor attributes and the sequence phylogeny, are given by a univariate leaf distribution, which is assumed to be the same for each individual. Four possible leaf distributions are defined: *Attraction*, having the predictor makes it more likely to have the target; *Repulsion*, not having the predictor makes it less likely to have the target; *Escape*, having the predictor makes it less likely to have the target; and *Reversion*, not having the predictor makes it more likely to have the target. The pair Attraction/Repulsion corresponds to a positive correlation between predictor and target, while the pair Escape/Reversion corresponds to a negative correlation between predictor and target.

## Results

### General clinical and geographical characteristics of the Mexican cohort

**Figure 1:**
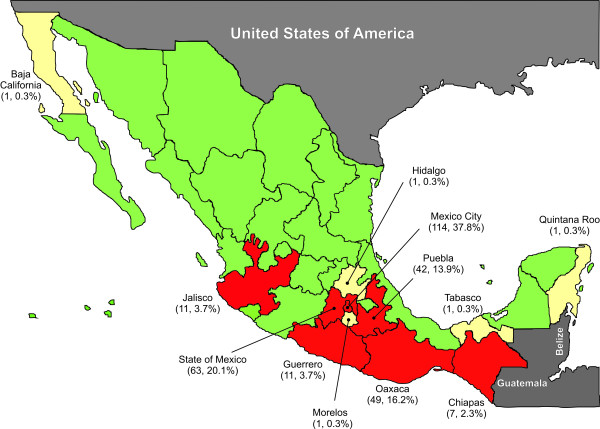
**Geographical residence of the individuals included in the study**. The map shows data for 302 antiretroviral treatment-naïve HIV-infected individuals. States in red account for 98.3% of the individuals included in the study. States in white account for 1.7% of the individuals of the cohort.

Figure 1 shows the geographical residence of the individuals included in the study. As is typical in Latin American cohorts [42,43], half of the individuals were found to be in relatively advanced stages of HIV infection (CD4+ T cell counts <200 cells/μL) at enrolment, with approximately half of these patients having less than 50 CD4+ T cells/μL. Only one of every 10 participating individuals was found to be at relatively early stages of the infection (CD4+ T cell count >500 cells/μL) (Table 1). Taking the cohort as a whole, the median CD4+ T cell count was lower than 200 cells/μL. The male-to-female ratio of infected individuals was 3 to 1 (Table 1), representing a slightly higher HIV prevalence among women than previously reported for the Mexican infected population [44], possibly suggesting a tendency towards increased HIV infection in females in the Latin American region [43]. A typical negative correlation was observed between CD4+ T cell counts and plasma viral loads (p < 0.0001), with a mean increase in viral load of 0.1 logarithms per 50 CD4+ T cell decrease. Taken together, these observations are representative of a typical Mexican cohort, comprised mainly of individuals in relatively advanced stages of HIV infection, often diagnosed at the moment of presentation at the health care centers due to AIDS-related opportunistic disease symptoms.

**Table 1:** Relevant clinical parameters for a cohort of 303 Mexican individuals.

**Clinical Parameters**	**Mean**	**Median**	**Standard Error**	**Standard Deviation**
CD4^+ ^T cell count (cells/μL)*	238.4	196,5	11.6	201.3
CD4+ T cell %*	14.0	12.0	0.6	10.4
Viral Load (RNA copies/mL)	233,528	105,000	16,927	292,691
Log Viral Load	4.917	5.021	0.044	0.767
CD4+ T cell count stratification* [n (%)]				
>500 cells/μL	35 (11.6%)		148 (49.3%)	
201 – 500 célls/μL	113 (37.7%)			
50 – 200 célls/μL	82 (27.3%)		152 (50.7%)	
<50 célls/μL	70 (23.3%)			
**Gender [n (%)]**				
Male	229 (75.6%)			
Female	74 (24.4%)			

*Data for 3 patients are missing

### HLA allelic and haplotypic frequencies in a cohort of HIV-positive Mexican individuals

292 HIV-positive individuals from Central/Southern Mexico for whom class I HLA-A, B and C typing was available were used to characterize the immunogenetic background of this cohort. HLA allelic frequencies for the Mexican cohort are shown in the Additional file 1: Figure S1, Table S1. The most frequent alleles at the HLA-A locus were A*02, A*24, A*68 and A*31; the most frequent alleles at the HLA-B locus were B*39, B*35, B*40 and B*15; and the most frequent alleles at the HLA-C locus were Cw*07, Cw*04, Cw*03 and Cw*08 (Additional file 1: Figure S1). Characteristically, more than 60% of the participating individuals expressed A*02, more than 50% expressed Cw*07 and more than a third expressed B*39 and/or B*35 (Additional file 1: Figure S1, Table S1).

**Figure 2:**
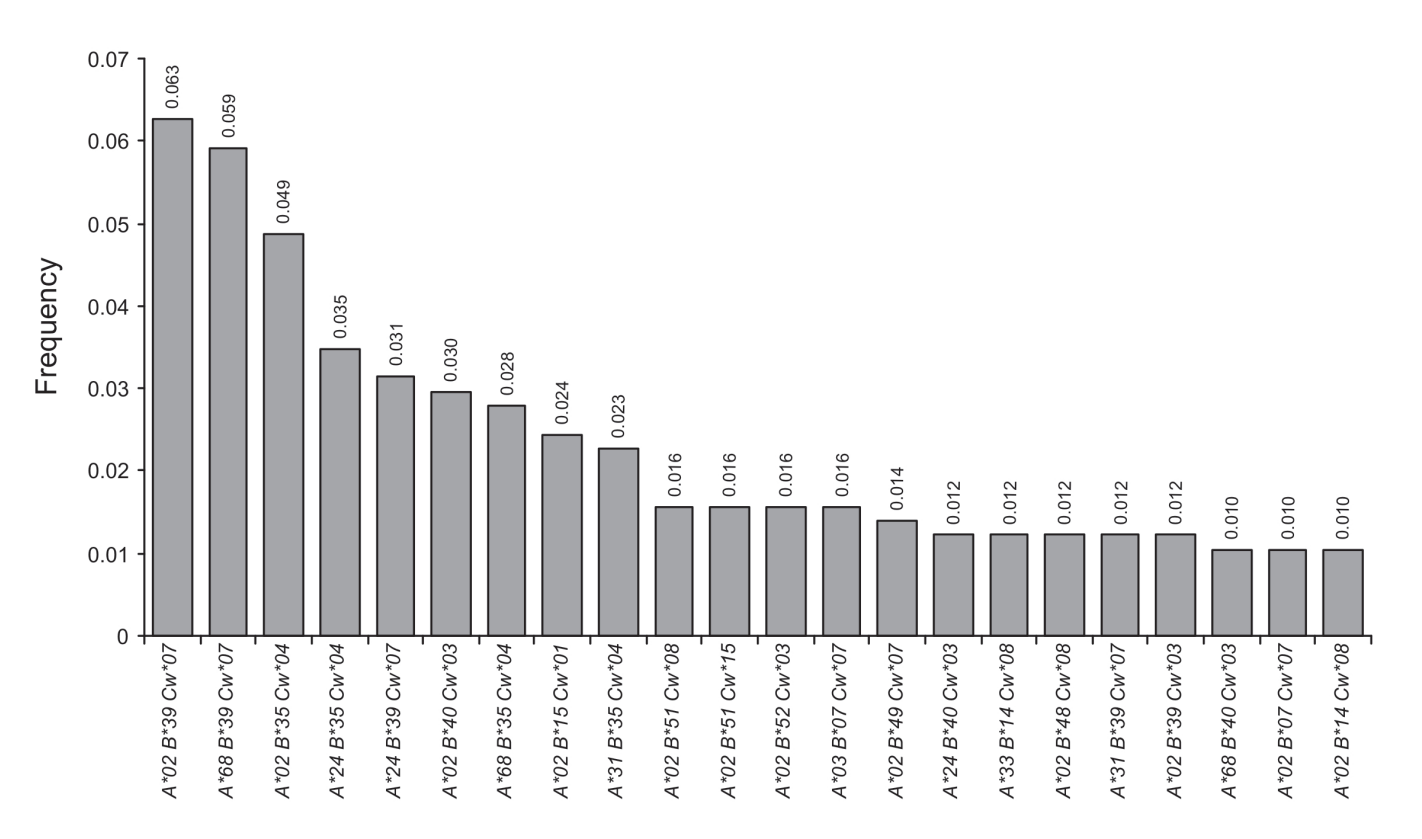
**Most frequent three-gene class I HLA haplotypes in a Mexican cohort of HIV-positive individuals**. Genetic frequencies were calculated for 292 HIV-positive individuals from Central/Southern Mexico. Gametic phase for each individual was estimated using the pseudo-Bayesian algorithm ELB, using the program Arlequin v3.11. HLA-A, B and C genes were typed at low/medium resolution by SSP-PCR as described in Methods. Haplotypes with frequencies over 1% in the cohort are shown.

In order to more precisely describe the immunogenetic background of the HIV-positive cohort of Mexican individuals, the frequencies of two and three-gene class I HLA haplotypes were estimated. Due to the fact that the cohort was composed of non-related individuals with unknown family genetic backgrounds, a gametic phase estimation for each individual was carried out prior to the calculation of HLA haplotype frequencies as described in the Methods. A total of 192 different three-gene HLA haplotypes were identified, of which 22 occurred at a frequency higher than 1% (Figure 2). The most frequent three-gene haplotypes were A*02/B*39/Cw*07, A*68/B*39/Cw*07 and A02*/B*35/Cw*04, all occurring at frequencies higher than 4.5%. Considering two loci, a total of 121 possible haplotypes were found for HLA-A/B, 82 for HLA-B/C and 92 for HLA-A/C. The most frequent two-gene haplotypes were A*02/B*39, A*02/B*35, A*24/B*35 and A*68/B*39 for HLA-A/B; B*39/Cw*07, B*35/Cw*04 and B*40/Cw*03 for HLA-B/C; and A*02/Cw*07, A*68/Cw*07 and A*02/Cw03 for HLA-A/C (Table 2). In general, there was lower variability among the HLA-B/C haplotypes compared to the HLA-A/C and the HLA-A/B haplotypes, possibly due to the frequent linkage disequilibrium observed between HLA-B and C genes (Additional file 1: Table S2).

**Table 2:** Most frequent two-gene class I HLA haplotypes in the cohort of HIV-positive Mexican individuals.†

**HLA A-B**	**Frequency**	**HLA B-Cw**	**Frequency**	**HLA A-Cw**	**Frequency**
A*02 B*39	0.0938	B*39 Cw*07	0.17422	A*02 Cw*07	0.10627
A*02 B*35	0.0486	B*35 Cw*04	0.14286	A*68 Cw*07	0.07840
A*24 B*35	0.0486	B*40 Cw*03	0.05401	A*02 Cw*03	0.07143
A*68 B*39	0.0486	B*07 Cw*07	0.04530	A*02 Cw*08	0.05226
A*02 B*51	0.0451	B*15 Cw*01	0.04530	A*02 Cw*04	0.04878
A*68 B*35	0.0451	B*14 Cw*08	0.03833	A*24 Cw*07	0.04355
A*02 B*40	0.0399	B*48 Cw*08	0.03659	A*02 Cw*01	0.03833
A*02 B*15	0.0330	B*44 Cw*16	0.02613	A*24 Cw*04	0.03833
A*31 B*35	0.0278	B*44 Cw*05	0.02265	A*31 Cw*04	0.02613
A*24 B*39	0.0243	B*49 Cw*07	0.02091	A*02 Cw*15	0.02265
A*03 B*07	0.0208	B*52 Cw*03	0.01916	A*68 Cw*04	0.02265
A*24 B*15	0.0208	B*51 Cw*15	0.01916	A*24 Cw*03	0.02265
A*02 B*52	0.0208	B*35 Cw*07	0.01742	A*01 Cw*07	0.02091
A*31 B*39	0.0191	B*08 Cw*07	0.01568	A*31 Cw*07	0.01916
A*02 B*48	0.0156	B*18 Cw*05	0.01568	A*68 Cw*03	0.01916
A*02 B*49	0.0139	B*51 Cw*08	0.01568	A*02 Cw*16	0.01568
A*24 B*40	0.0139	B*38 Cw*12	0.01394	A*03 Cw*07	0.01568
A*30 B*18	0.0122	B*39 Cw*03	0.01394	A*24 Cw*08	0.01394
A*68 B*40	0.0122	B*35 Cw*03	0.01045	A*33 Cw*08	0.01394
A*24 B*44	0.0122	B*52 Cw*12	0.01045	A*29 Cw*16	0.01220
A*29 B*44	0.0122	B*55 Cw*07	0.01045	A*30 Cw*05	0.01045
A*33 B*14	0.0122			A*31 Cw*01	0.01045
A*01 B*57	0.0104			A*24 Cw*05	0.01045
A*02 B*07	0.0104				
A*02 B*44	0.0104				
A*02 B*55	0.0104				

†292 HIV positive individuals from Central/Southern Mexico were included. Gametic phase for each individual was estimated with the Arlequin v3.11 software as described in the Methods. Haplotypes with frequencies over 1% are shown.

**Figure 3:**
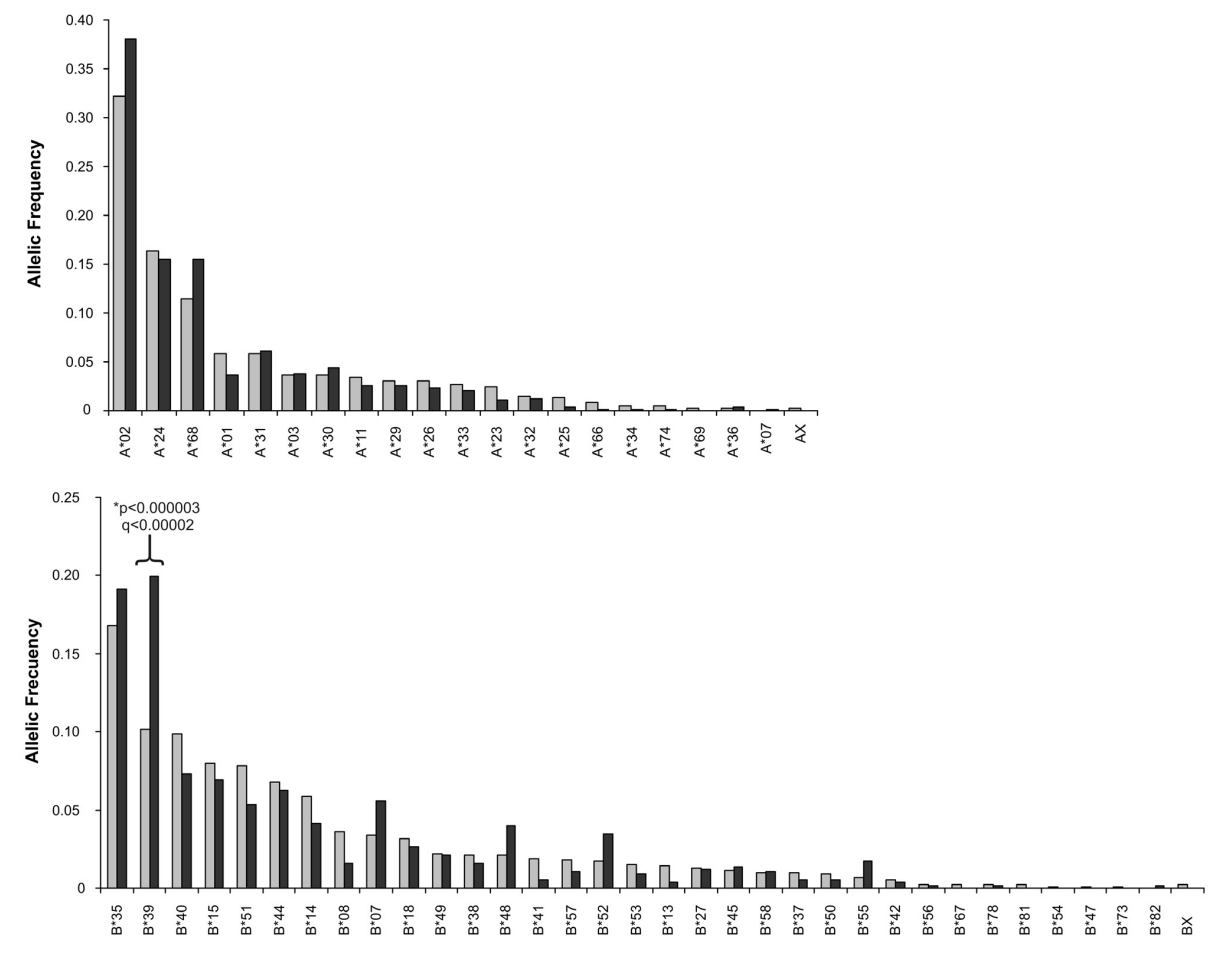
**Differences in HLA frequencies between HIV-positive and HIV-negative Mexicans**. Allelic frequencies were calculated for HLA-A and B genes, in the cohort of 292 HIV-positive individuals from this study (dark grey) and compared to those previously reported for a cohort of 381 individuals of 191 Mexican families by Barquera et al [38] (light grey). HLA typing in both cases was carried out by SSP-PCR as described in the Methods. For comparability, HLA nomenclature for histocompatibility used by Barquera et al. was substituted with its genetic equivalent, i.e. B65 and B64 were included as B*14 alleles; B62, B63, B70, B71, B72, and B75 were included as B*15 alleles; and B61, and B60 were included as B*40 alleles, according to the equivalents accepted by the WHO Nomenclature Committee for Factors of the HLA System .

HLA-A and B allelic frequencies in this study were compared to those previously reported in an open population-based study of 381 individuals from 191 Mexican families from Central/Northern Mexico [38] (Figure 3). Although the geographical origin of the individuals in the latter study differs somewhat from that of the individuals in the present study, the large number of individuals from the Central part of Mexico and the fact that the HLA typing method used was the same as ours, renders this study an adequate reference for a typical HIV-negative population in Mexico for comparison with our study. The HLA frequency distribution of loci A and B was significantly different between the two studies (chi^2 ^= 99.39, p = 0.00008), with differences in residuals seen only in B*39 (p = 2.25E-06, q = 1.19E-04), a typical Amerindian allele group, which showed a frequency nearly two-fold higher in HIV-positive individuals compared to HIV-negative individuals (Figure 3). Whether having B*39 represents a risk factor for HIV infection in Mexico remains to be confirmed, as the high frequency of this allele could also reflect an epidemiological phenomenon such as B*39 being enriched in the most affected sectors of the population by HIV infection or simply be a sample bias of the individuals included in either of the two studies.

To our knowledge, this study is the first formal report of class I HLA frequencies in a typical HIV-infected Mexican cohort.

### Unique immunogenetic Background in a cohort of HIV-infected, antiretroviral treatment naïve individuals from Central/Southern Mexico

**Figure 4:**
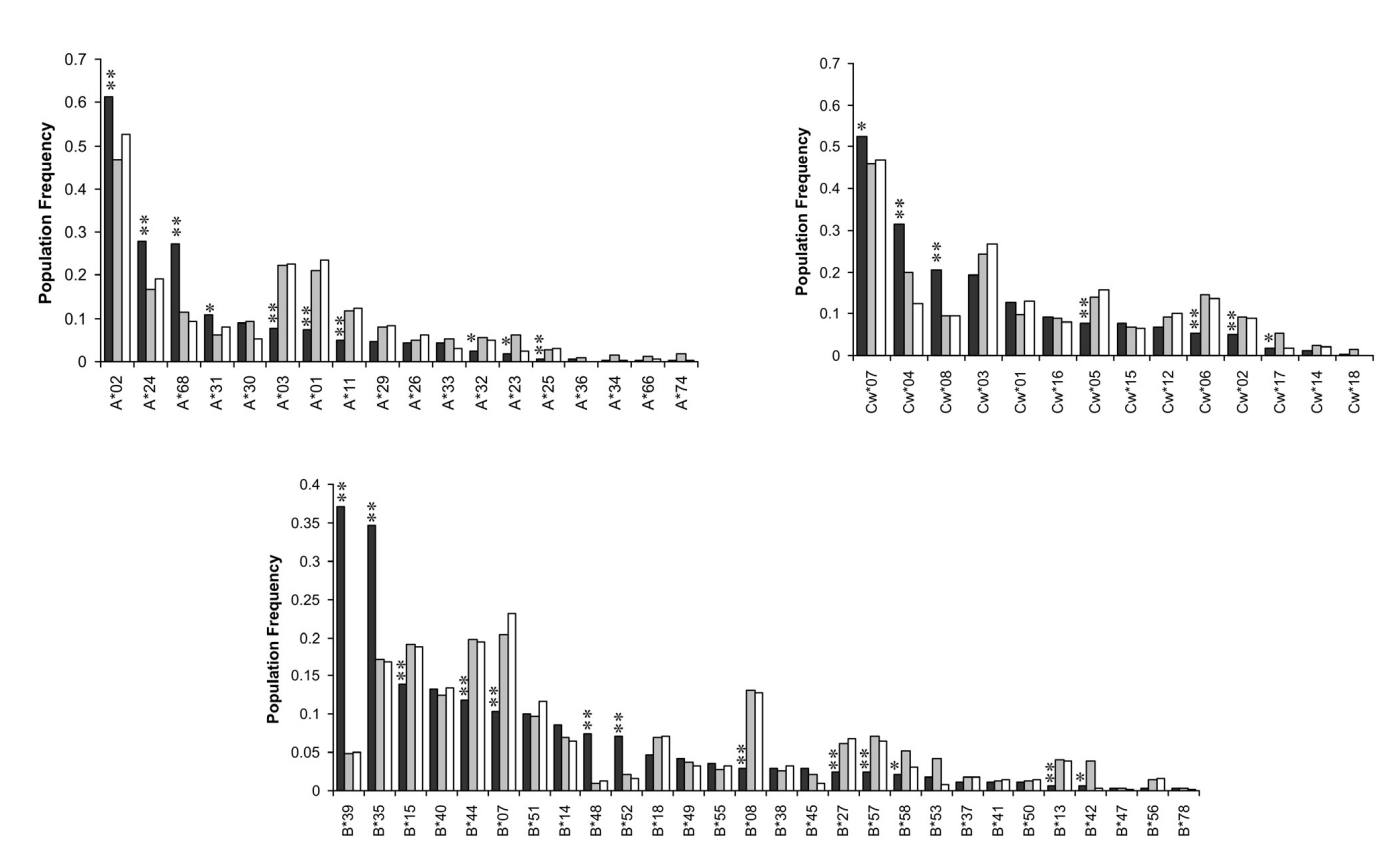
**Marked differences in HLA allele frequencies in three clade B-infected cohorts**. Population frequencies for class I HLA genes A, B and C were compared between the Mexican cohort described in this study (n = 292) (dark grey). The combined IHAC cohort including individuals from British Columbia, Canada; Western Australia and the USA (n = 1845) (light grey) [37] (Brumme ZL, John M, et al, PLoS ONE 2009, in press), and the British Columbia HOMER cohort (n = 1045) (white) described in detail previously [34]. **Significant differences (q < 0.05) between the Mexican cohorts and both the IHAC and the HOMER cohorts, *significant differences (q < 0.05) between the Mexican cohort and the IHAC cohort only.

In order to highlight the unique immunogenetic background of the Mexican population with respect to other populations in which HLA-associated HIV evolution has been studied, an HLA frequency comparison was carried out between our cohort of 292 HIV-positive individuals from Central/Southern Mexico, a previously described cohort of 1,045 HIV-positive individuals from British Columbia, Canada (HOMER cohort) [34] and the large International HIV Adaptation Combined (IHAC) cohort, including 1,845 individuals from British Columbia, Canada; Western Australia and the USA (Figure 4). Although both the HOMER cohort and the USA subset of the IHAC cohort include a minority of individuals self-identified as Hispanic, important differences were seen in HLA allele distribution in the three cohorts that account for the typical genetic admixture of the Mexican population [38,39]. As expected, there were significant differences between the allele frequencies of the cohort reported here and the HOMER and IHAC cohorts (chi^2 ^= 597.41 and 782.13, p < 10^-88 ^and 10^-125^, respectively). HLA-A*68, B*35, B*39, B*48, B*52, Cw*04 and Cw*08 alleles were observed at significantly higher frequencies in the Mexican cohort compared to HOMER and IHAC cohorts (p < 0.005, q < 0.01), consistent with typical Amerindian alleles [38,39,45]. Similarly, HLA-A*01, A*03, A*11, B*07, B*08, B*13, B*27, B*44, B*57, Cw*05, and Cw*06 alleles were observed at significantly lower frequencies in the Mexican cohort compared to HOMER and IHAC cohorts (p < 0.005, q < 0.01), consistent with the higher frequency of these alleles among Caucasians [38,39,45] (Figure 4). Additionally, HLA-A*02 and A*24 alleles had significantly higher frequencies, and HLA-A*25, B*15 and Cw*02 alleles had significantly lower frequencies in the Mexican cohort than in HOMER and IHAC cohorts, not specifically reported to be enriched in Amerindian, or Caucasian groups. Notably, the frequency of HLA-B*39 alleles was more than 7 times higher in the Mexican cohort than in HOMER and IHAC cohorts (Figure 4). Taken together, these results confirm the characteristic admixture of the mainly Amerindian and Caucasian genes of the Mexican mestizo population in a typical cohort of HIV-infected individuals from the Central/Southern region of the country, and reveal a previously uncharacterized, unique immunogenetic background for the study of HLA-associated HIV evolution at the population level.

### HLA-mediated HIV evolution in a Mexican cohort

HIV evolution mediated by HLA selection at the population level was studied using a 434 amino acid fragment spanning the whole HIV protease and 335 codons of the reverse transcriptase in 280 chronically-infected individuals from this cohort. The phylogenetic dependency network (PDN) model by Carlson et al [14], currently one of the most comprehensive models to assess HLA-mediated HIV evolution, was applied to infer patterns of CTL escape and codon co-variation in the Mexican cohort. Our results were compared with those previously derived from applying the PDN model to a thoroughly characterized, multi-center, combined cohort of predominantly clade B-infected, antiretroviral treatment-naïve individuals from British Columbia, Canada; Western Australia; and the USA (the IHAC cohort), with a clearly different immunogenetic background compared to the Mexican cohort [14,34,37,46] (Figure 4). The Mexican cohort was also found to be predominantly clade B-infected (99.64%) with only one subtype other than B/recombinant form (CRF_06_cpx) identified (REGA HIV-1 Subtyping Tool 2.0, ). A phylogenetic tree for the Mexican *pol *sequences included in this study is shown in the Additional file 1: Figure S2.

**Figure 5:**
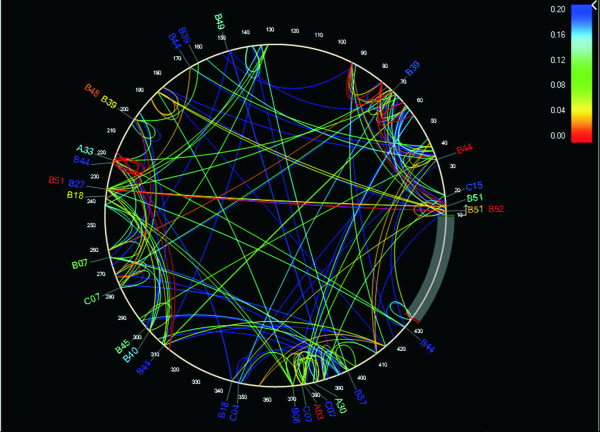
**Protease and RT phylogenetic dependency network for the Mexican cohort**. A phylogenetic dependency network map was generated with the PhyloDv software . Pol positions are drawn counter clockwise, with the N-terminus of the protease at the 3 o'clock position, and the first RT codon corresponding to position 100. Lines indicate associations between codons (inside the circle) or between HLA alleles and codons (outside the circle). Colors indicate q-values of the most significant associations between two attributes. Associations with q < 0.2 for 280 individuals from Central/Southern Mexico were included.

The PDN model was used to identify significant HLA-HIV codon as well as HIV codon-HIV codon associations, using a q-value threshold of 0.2. Due to the fact that the PDN model uses a multivariate model in which several predictor attributes (i.e. the presence or absence of a specific HLA or amino acid at an HIV codon) can be associated with the presence or absence of a specific amino acid at an HIV target codon, spurious associations explained by the presence of linkage disequilibrium among HLA alleles and HIV codon covariation were minimized. A total of 43 HLA-HIV codon and 251 HIV codon-HIV codon associations were identified, representing 30 different HLA-HIV codon and 135 HIV codon-HIV codon pairs (Additional file 1: Table S3). This association network was depicted graphically with the PDN viewer PhyloDv [14] (Figure 5), showing the HIV amino acid sequence as a circle with lines joining HLA alleles and associated HIV codons outside the circle and arcs joining covarying HIV codons within the circle. Even with a relatively small number of individuals in the cohort, a dense association network was observed at q < 0.2 that reveals characteristic patterns of HIV codon covariation and HLA-mediated substitutions in the studied cohort. HLA associations were found at 6.1% of protease codons, and at 7.1% of RT codons. As previously described for HIV Gag [14], covarying codons were more frequent within a sub-protein (75.6% total: 20% within the protease and 55.6% within the reverse transcriptase) than between sub-proteins (24.4%; p < 0.001). 28/135 (20.7%) of HIV codon pairs were within 10 positions of each other, suggesting a close proximity in an important proportion of compensatory mutations, or the targeting of multiple epitopes by the same HLA allele. Notably, 46.7% of HLA-HIV codon associations predicted substitutions at other codons, suggesting complex HLA-mediated escape pathways.

Interestingly, there were only two HIV *pol *sites previously associated with resistance to antiretroviral drugs that were also predicted to be associated with HLA selective pressure. B*18 was associated with an E to A change in RT position 138. The polymorphism 138A is associated with decreased response to non-nucleoside RT inhibitors (NNRTIs), including etravirine (Stanford University HIV Drug Resistance Database, ). Similarly, Cw*07 was associated with a lower probability of having a D residue and a tendency for conservation of a V residue in RT position 179. The polymorphism 179D is associated with low level resistance to NNRTIs (Stanford University HIV Drug Resistance Database, ). These observations show that HLA-mediated evolution can influence antiretroviral drug resistance, both promoting and preventing the presence of drug-resistance-related polymorphisms. This dual pressure phenomenon has been described previously [35,47]; however, its frequency and population impact in the Mexican cohort will have to be assessed further.

**Figure 6:**
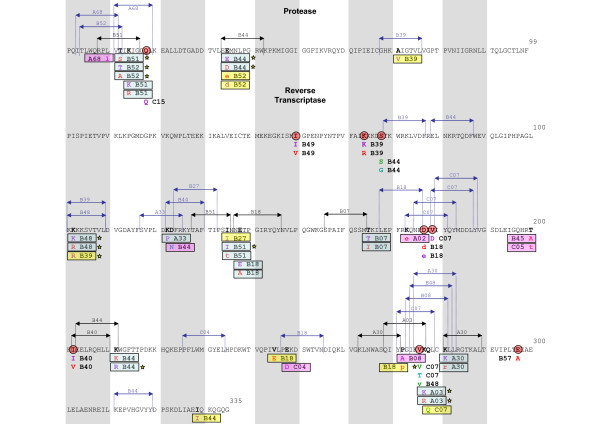
**Epitope map supporting HLA-HIV polymorphism associations obtained by the Phylogenetic Dependency Network model in the Mexican cohort**. HLA-HIV polymorphism associations were searched in *pol *sequences derived from 280 individuals from Central/Southern Mexico using the previously described PDN model [14] that corrects for the confounding effects of HIV phylogeny, HIV codon covariation, and linkage disequilibrium of HLA alleles. Significant HLA-HIV polymorphism associations (q < 0.2) were located in an epitope map. Strongly significant associations (q < 0.05) are marked with yellow stars. Experimentally defined CTL epitopes are shown in black http://www.hiv.lanl.gov/content/immunology/index.html, epitopes predicted by HLA peptide binding motifs are shown in blue (Motif Scan, Los Alamos HIV Database, http://www.hiv.lanl.gov/content/immunology/tools-links.html ). Target HIV positions are shown in bold, including the predictor HLA allele and the associated amino acid residue in different colors depending on the type of leaf distribution of the association obtained with the PDN model, i.e. escape (purple; having the predictor HLA makes it less likely to have the target amino acid), attraction (red; having the predictor HLA makes it more likely to have the target amino acid), reversion (green, not having the predictor HLA makes it more likely to have the target amino acid), repulsion (light blue; not having the predictor HLA makes it less likely to have the target amino acid). Amino acids in lower case indicate associations obtained without considering codon covariation. HLA-HIV polymorphism associations in the Mexican cohort were compared to the ones observed in the multicenter IHAC cohort including 1845 individuals from British Columbia, Canada, the USA and Western Australia [37] (Brumme ZL, John M, et al, PLoS ONE 2009, in press). Shared associations between the Mexican and IHAC cohorts are shown in blue rectangles; shared target HIV positions with shared target amino acids that were associated with different predictor HLA alleles in each cohort are shown in yellow rectangles; shared target positions with different HLA predictors and different associated target amino acids are shown in pink rectangles. HLA-associated HIV positions observed exclusively in the Mexican cohort are marked with red circles.

HLA-HIV codon associations found for the Mexican cohort at q < 0.2 are presented in an epitope map in order to confirm the validity of the associations (Figure 6). 10 HLA-HIV codon pairs can be explained by experimentally confirmed epitopes, of which 5 have been optimally defined (Los Alamos HIV Database, ). Twelve additional HLA-HIV codon pairs can be confirmed by epitope prediction with HLA peptide binding motifs (Motif Scan Tool, Los Alamos HIV Database, ). Eight HLA-epitope pairs could not be explained by epitope mapping, possibly because of lack of data on peptide binding motifs of associated HLA alleles (e.g. B*39 and B*49), or because of the presence of false positive associations (at q < 0.2, we expect 20% of the associations to be false positives). The possibility also exists that these associations represent escape mutations within unusual (novel) epitopes, or escape mutations that influence epitope processing that may occur far away from the actual epitope. Indirect or "one-hop" associations of the type a->b->c, where the HLA allele "a" is shown to predict the polymorphism "c", would be improbable as the multivariate model of the PDN model minimizes them. The same is true for associations with alleles in linkage disequilibrium with the selective allele, as linkage disequilibrium is accounted for by the PDN model. Some additional associations observed without taking codon covariation into account are also shown (Figure 6). Not considering codon co-variation increases the power to detect associations, but allows the presence of indirect associations.

HLA-HIV codon associations found in the Mexican cohort were compared to the ones previously found in a dataset 1845 HIV *pol *sequences from the combined IHAC cohort [37] (Brumme ZL, John M, et al, PLoS ONE 2009, in press) (Figure 6, Additional file 1: Table S3). Finding HLA-HIV codon associations in the smaller Mexican cohort that were not observed in the well-powered IHAC cohort could be indicative of unique HLA-driven HIV evolution in immunogenetically distinct cohorts. Not surprisingly, many of the observed HLA-HIV codon associations in the Mexican cohort were also predicted in the IHAC cohort, supporting the observation of highly conserved mutational patterns in HLA-driven HIV evolution (Figure 6, Additional file 1: Table S3). Nevertheless, important differences were also noted between the two cohorts. From the 43 HLA-HIV codon associations observed in the Mexican cohort, 23 were identified as novel associations, not previously observed in the larger IHAC cohort (nor in previous similar studies [25,30,34]), representing 18 different HLA-HIV codon pairs. Although several of these are likely to be false positives due to the 20% FDR, the fact that 53% of the present associations were not found in the well-powered IHAC cohort is striking. Furthermore, of these 18 new HLA-HIV codon pairs, 8 involved associations with codons that were not associated with any HLA allele in the IHAC cohort, and 4 involved associations with B*39, which is substantially more frequent in the Mexican cohort than in the IHAC cohort. Remarkably, 17 of the 23 novel associations involved HLA alleles whose frequencies were statistically indistinguishable from those in IHAC, suggesting that their presence is not due to increased statistical power, but rather may be due to differences in patterns of epitope targeting. In addition, 2 of the novel associations involved B*27 or B*08, two alleles that were significantly less frequent in the present cohort than in IHAC, which may reflect differences in epitope targeting between the cohorts or the fixation (and resulting drop in statistical power) of escape mutations in the IHAC cohort [9]. Interestingly, although previously identified as HLA-associated in the IHAC cohort, some HLA-associated HIV codons in the Mexican cohort showed different HLA specificities and/or target amino acids. This was the case in 13 of the 23 novel HLA-HIV codon associations (10 of 18 HLA-HIV codon pairs). For example, Protease 71 V was associated with B*39 in the Mexican cohort, while it was associated with B*15 in the IHAC cohort; RT 245E was associated with B*18 in the Mexican cohort, but with B*57 in the IHAC cohort. Not surprisingly, B*39 was much more frequent in the Mexican cohort (p = 1.80E-44, q = 1.21E-42), while B*15 was more frequent in the IHAC cohort (p = 0.00964, q = 0.0231) (Figure 4). On the other hand B*57 was less frequent in the Mexican cohort (p = 0.000430, q = 0.00180) and no significant difference was found for B*18. These associations can be explained by experimentally confirmed or predicted epitopes both in the Mexican cohort and in the IHAC cohort (Figure 6; Los Alamos HIV Immunology Database, ). Both B*15 and B*39 are predicted to have an epitope in position PR 68–76. These observations support the existence of sites in the HIV genome whose sequence variability at the population level reflects active selection pressure by different HLA alleles, and support previous observations that different HLA alleles may drive identical (as well as conflicting) escape mutations [48].

Interestingly, different consensus amino acids between the Mexican and the HOMER cohorts were detected at four Pol codons (Table 3). Two of these sites were HLA-associated in both cohorts, (RT 272 and RT 277), one was HLA-associated in only the HOMER cohort (PR 93) and one was not found to be associated to HLA in either cohort (RT 293) (although the possibility of an undetected association with HLA cannot be discarded). This observation supports the possibility of finding different HLA footprints in different populations, even between cohorts predominantly infected with viruses of the same clade. The finding that three of the four observed changes in consensus amino acids between the Mexican and the HOMER cohorts could have originated from HLA-driven pressure is also noteworthy. The role of HLA frequency in the fixation of escape mutations was evident in the A*03-associated position RT 277, where the escaped form R has become fixed in the HOMER Pol consensus, while mainly remained as the susceptible form K in the Mexican Pol consensus. The A*03 allelic frequency in the HOMER cohort was three times higher than in the Mexican cohort (p = 7.08E-10, q = 1.00E-08) (Table 3, Figure 4).

**Table 3:** Point differences in consensus sequences between the Mexican and the HOMER cohorts.

HIV position	Susceptible	Escaped	Mexican Consensus	HOMER Consensus	B Consensus	Associated HLA Mexican cohort	Associated HLA HOMER cohort	HLA frequency Mexican cohort	HLA frequency HOMER cohort
PR 93	I	L	L	I	I	-	B*15	0.0702	0.1131
RT 211	R	G, K	K	K	R	B*44	B*44, B*15	0.0616†	0.1059†
RT 272	P, A	S	P	A	A	B*08	B*42	0.0154†	0.0693†
RT 277	K	R	K	R	K	A*03	A*03	0.0411	0.1235
RT 293	-	-	I	V	I	-	-	-	-

†Frequency of the associated HLA allele in the Mexican cohort is shown.

As previously observed [22,49], HLA-B alleles were involved in the majority of the associations (73% of HLA-HIV codon pairs), compared to HLA-A and C alleles in the Mexican cohort (p < 0.001 for both cases). Interestingly, 16.6% of HLA-HIV codon pairs were due to HLA-C alleles. Although in previous studies many of the associations apparently defined by HLA-C alleles represented indirect associations with HLA-B or A alleles due to the HLA linkage disequilibrium phenomenon, the PDN model used in the present study accounts for HLA linkage disequilibrium, minimizing the risk of finding these and other kinds of indirect associations. Nevertheless, it is important to note, that the ability of the PDN model to correct for HLA linkage disequilibrium is positively correlated with sample size and negatively correlated with the strength of linkage disequilibrium. Thus, false positive associations could still be found when strong linkage disequilibrium patterns exist, and random noise makes it difficult to distinguish the true associations. This could be the case for Cw*15-associated position PR 18, which could not be explained by epitope prediction. Cw*15 and B*51 are in strong linkage disequilibrium in the Mexican cohort (Additional file 1: Table S2), and an experimentally confirmed B*51 epitope exists that could explain the association. Although many of the HLA-C associations had high q-values, and could represent false positive associations, some of them were strongly associated and were in consonance with predicted and verified epitope mapping (Additional file 1: Table S3, Figure 6). These observations suggest an important role of HLA-C alleles in shaping HIV evolution at the population level in the Mexican cohort.

On the other hand, B*44 alone was responsible for 16.6% of HLA-HIV codon pairs, followed by B*51 and B*39, each responsible for 10% of the observed HLA-HIV codon pairs (Additional file 1: Table S3, Figure 6). This predominance is not observed in the IHAC cohort, possibly suggesting different patterns of immunodominance and HIV immune escape resulting from different epitope targeting between the two cohorts. These observations further support the existence of differential patterns of HIV selection by HLA alleles in populations worldwide.

Taken together, these results support the existence of highly conserved, universal HLA-mediated mutational patterns or "footprints" on HIV sequences at the population level. However, they also suggest that unique characteristics could exist in HLA-mediated HIV evolution in immunogenetically distinct populations, which can be detected even with cohorts of relatively small number of individuals.

### Differences in HIV evolution between free plasma virus and PBMC proviral sequences

**Figure 7:**
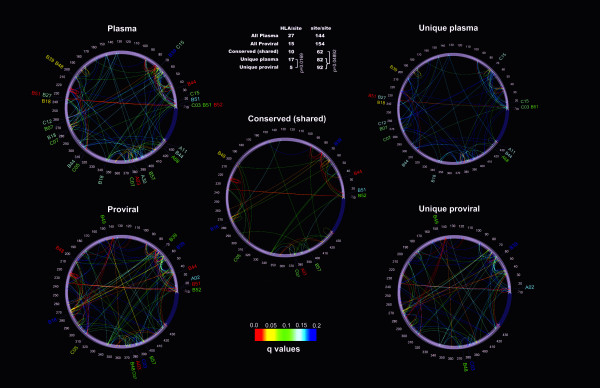
**HLA-HIV codon and HIV codon-HIV codon associations for the free plasma virus and PBMC proviral Pol sequences**. Phylogenetic dependency network maps were generated with the PhyloDv software . Each map shows HLA associations as tags pointing to their corresponding sites, and HIV codon-HIV codon associations as inner arcs connecting the associated sites. Plasma: all associations in free plasma virus from 250 patients that had a corresponding PBMC proviral sequence. Proviral: all associations for 250 PBMC proviral sequences. Conserved (shared): shared HLA-site or site-site associations between proviral and plasma sequences. If one amino acid at one site was associated with two different amino acids at another site (e.g. one for escape, one for reversion), only one association was counted. Unique plasma: associations only found to be significant in free plasma virus samples. Unique proviral: Associations only found to be significant in PBMC proviral samples. Significance was calculated with the binomial test; q-values are represented as a heat map shown in the lower inset. Associations with q < 0.2 are shown (all associations had p < 0.002). The upper inset table shows the number of significant associations in each map.

A subset of 250 HIV-infected individuals for whom HLA typing and both *pol *PBMC proviral sequences and free plasma virus *pol *RNA sequences were available was used to compare HLA associated polymorphisms in two different viral compartments at the population level. The PDN model was applied to both sequence datasets and results were graphically depicted with the PDN viewer PhyloDv [14] (Figure 7). In all, 36 HLA-HIV codon associations were found for the free plasma virus sequences and only 24 for the PBMC proviral sequences, representing 27 and 15 HLA-HIV codon pairs respectively (Figure 7, Table 4). Interestingly, the number of unique HLA-HIV codon pairs observed in free plasma virus sequences was significantly higher than the number of unique pairs in proviral sequences (p = 0.0169), and only 10 of the HLA-HIV codon pairs were observed in the two viral compartments. These results are consistent with a recent study that reported the presence of HLA-associated escape mutations in plasma sequences that were rarely seen in the proviral population within some infected individuals [50]. The observation that an overall different evolution was seen in plasma viral sequences compared to proviral sequences, with a significantly lower number of HLA-associated sites in proviral sequences, is consistent with the model that suggests that proviral sequences represent early archived HIV in the latent reservoir and that plasma sequences represent a population that has evolved further in response to immune selective pressure. Furthermore, these observations are suggestive of a dynamic development of CTL responses throughout the infection, such that early CTL responses are reflected in the archival proviral compartment while the plasma compartment reflects more recent CTL responses [51,52]. We note, however, that proviral HLA-HIV site associations did not correlate with previously defined rapidly escaping sites under HLA pressure in clade B-infected Caucasian individuals [25]. This fact might reflect different rates of escape between demographically divergent cohorts, or it might reflect differing compartment-based CTL selective pressures that are simply reflective of the archival nature of the proviral sequences.

**Table 4:** Protease and Reverse Transcriptase HLA-HIV codon associations in free plasma virus RNA and PBMC proviral DNA for the Mexican cohort.

**Free plasma virus *pol *sequences†**						**PBMC proviral *pol *sequences†**					
HLA	Associated HIV Codon		Conditioning variables	p-value	q-value	HLA	Associated HIV codon		Conditioning variables	p-value	q-value
						A*02^c^	PR	15I	PR 77V^c^, PR 36M^d^, PR 13I^a^	0,0010442	0,1522376
A*03^b^	RT	277R		1,14E-08	1,10E-05	A*03^b^	RT	277R		1,74E-09	1,48E-05
A*03^a^	RT	277K		1,14E-08	1,10E-05	A*03^a^	RT	277K		1,74E-09	1,48E-05
A*11^b^	RT	333E	PR 35E^c^, RT 293I^a^, RT 207Q^d^, PR 62I^d^	0,00113806	0,1336789						
A*30^c^	RT	281K	RT 278Q^d^	0,00109204	0,1336789						
A*68^a^	RT	326V		0,00035689	0,1048983						
B*07^b^	RT	165I		0,00067347	0,1191747						
B*07^a^	RT	165T		0,00067347	0,1191747						
B*18^b^	RT	138A		0,00012948	0,0494756						
B*18^a^	RT	138E		0,00012948	0,0494756						
B*18^b^	RT	245E	PR 45K^c^	0,00068267	0,1191747						
B*18^b^	RT	177D		0,00094587	0,1312293	B*18^b^	RT	177D	RT 123D^d^, RT 121D ^d^	0,0017257	0,19555
B*18^a^	RT	177E		0,00094513	0,1312293	B*18^a^	RT	177E		0,0006388	0,1183628
B*18^a^	RT	245V		0,00115452	0,1336789						
B*27^a^	RT	135I	B*51^a^, PR 12T^d^, RT 202I^d^	0,00096164	0,1312293						
B*39^a^	RT	102R	B*48^b^, RT 64K^a^	0,00016533	0,0539628						
B*39^c^	PR	71V	PR 93L^d^, PR 39P^a^, PR 57K^d^, Cw*15^b^	0,00193116	0,1994313	B*39^c^	PR	71V	PR 57R^a^, PR 93I^c^, Cw*15^d^, RT 178M^b^, PR 39P^a^	0,0003592	0,0888581
						B*39^a^	PR	60E	PR 39Q^b^, PR 37D^b^, PR 37E^b^	0,0019258	0,199068
B*44^b^	PR	35D		1,16E-08	1,10E-05	B*44^b^	PR	35D		5,77E-08	0,0002091
B*44^a^	PR	35E		1,16E-08	1,10E-05	B*44^a^	PR	35E		5,77E-08	0,0002091
B*44^a^	RT	211R		0,00112279	0,1336789						
B*44^a^	RT	329I	RT 334L^b^	0,00120957	0,1359348						
B*48^b^	RT	102R		2,47E-08	1,89E-05	B*48^b^	RT	102R		1,53E-11	3,88E-07
B*48^a^	RT	102K		0,00016947	0,0539628	B*48^a^	RT	102K		1,90E-05	0,0120849
						B*48^a^	RT	276V	RT 272P^c^, Cw*07^c^	0,0005419	0,1049938
						B*49^a^	RT	48S		0,0004979	0,1035941
						B*49^b^	RT	48T		0,0004979	0,1035941
B*51 ^a^	RT	135I		4,03E-07	0,0002198						
B*51^b^	PR	12S		0,00038855	0,1060476						
B*51^b^	PR	14R	RT 135I^a^, RT 200A ^a^	0,00144043	0,1528856	B*51^b^	PR	14R		7,41E-06	0,005226
B*51 ^a^	PR	14K	RT 135I^b^, RT 200A^b^	0,00138006	0,1506635	B*51^a^	PR	14K		7,41E-06	0,005226
B*52^b^	PR	12A		5,59E-07	0,0002671	B*52^b^	PR	12A	RT 135T^d^	0,0005416	0,1049938
B*52 ^a^	PR	12T		3,73E-08	2,38E-05	B*52^a^	PR	12T		1,60E-06	0,0016939
B*57^b^	RT	297A		0,00060553	0,1191747	B*57^b^	RT	297A		0,0004121	0,0941468
						Cw*03^b^	RT	283I		0,0017747	0,1964929
						Cw*03^a^	RT	283L		0,0017747	0,1964929
Cw*03^b^	PR	12P	PR 14R^d^, PR 67C^c^	0,00070538	0,1191747						
Cw*05^b^	RT	214L	RT 118I^b^	0,00065426	0,1191747	Cw*05^b^	RT	214L	RT 118V^c^	0,0002149	0,0665192
Cw*05 ^a^	RT	214F	RT 118I^a^	0,00065426	0,1191747	Cw*05^a^	RT	214F	RT 118V^d^	0,0002149	0,0665192
Cw*07^c^	RT	276V	RT 272P^c^	0,00054742	0,1191747	Cw*07^c^	RT	276V	RT 272P^c^	0,0004154	0,0941468
Cw*07 ^a^	RT	179D		0,00072749	0,1191747						
Cw*12^b^	RT	162S	PR 64L^b^, RT 165T^c^, PR 91T^c^, RT 135V^a^	0,00106536	0,1336789						
Cw*15^a^	PR	18Q		0,00074855	0,1191747						
Cw*15^b^	PR	71V	PR 93L^d^, PR 39P^a^, PR 57K^d^	0,0009574	0,1312293						

†Associations for 250 plasma virus sequences and the corresponding PBMC proviral sequences with q < 0.2 are shown. ^a ^escape (having the predictor makes it less likely to have the target), ^b ^attraction (having the predictor makes it more likely to have the target), ^c ^reversion (not having the predictor makes it more likely to have the target), ^d ^repulsion (not having the predictor makes it less likely to have the target.

Interestingly, the number of covarying HIV sites common to both compartments was lower than the number of covarying sites observed exclusively in plasma or in proviral sequences (p = 0.0489). Moreover, a large number of covarying sites was seen in proviral HIV sequences possibly reflecting remnants of viral adaptation to previous hosts.

Overall, differences in HLA-mediated selection were observed in the plasma virus and the PBMC provirus compartments, suggesting a highly dynamic HLA-associated evolution in HIV, as many of the HLA-HIV codon associations in the free plasma virus compartment were not evident in the proviral dataset, which likely contains early archived HIV sequences that appear to reflect less adaptation to within host HLA-mediated immune responses.

## Discussion

In this study we have presented evidence suggesting that a unique HLA allele frequency distribution in a cohort of clade B-infected Mexican individuals has left unique footprints on HIV sequences at the population level. We studied HLA-mediated HIV evolution in a clade B-infected Mexican cohort, comparing our data with data from the IHAC cohort, the largest clade B-infected cohort used to assess HLA-mediated evolution so far, which is composed of individuals from Canada, Australia, and the USA [37] (Brumme ZL, John M, et al, PLoS ONE 2009, in press). The two cohorts were shown to present notably different immunogenetic backgrounds, with an important admixture of Amerindian genes in the population of the Central/Southern part of Mexico (Figure 4). These different immunogenetic backgrounds provided a chance to assess the role of different HLA allele distributions in HLA-mediated selection in two cohorts infected by viruses of the same clade. The present cohort was shown to reflect the typical characteristics of an HIV-infected Mexican cohort, enriched in individuals in relatively advanced stages of HIV disease and presenting a similar HLA allele frequency distribution to the general population (Figure 3), with some specific exceptions (e. g. B*39) that will have to be assessed further in future studies.

Previous studies have suggested that HIV evolution at the population level follows broadly predictable, highly conserved mutational patterns associated with host CTL selective pressure [16,23,24,33-35]. Conclusions obtained from a direct comparison between different studies in different populations have been limited mainly due to the use of different methods and models for assessing HLA-mediated viral evolution in which important sources of confounding are frequently not accounted. We applied the recently described PDN model [14], which simultaneously accounts for HLA linkage disequilibrium, HIV codon co-variation and viral lineage effects, to clade B *pol *sequences (Additional file 1: Figure S2) from the present cohort and compared the results to the immunogenetically distinct population of the IHAC cohort. Our data support the observations of highly conserved, universal, HLA-associated footprints in the HIV proteome at the population level, as many of the HLA – HIV codon associations found in the Mexican cohort have consistently been observed in the IHAC cohort, as well as in previous studies with diverse cohorts [24,33-35,37].

Interestingly, however, our data also suggest the existence of unique HLA-associated footprints in HIV, which could be influenced by specific HLA frequency distributions in different HIV-infected populations. The unique characteristics of HLA-mediated selection in the Mexican cohort was revealed not only by the presence of unique HLA-HIV codon pairs not detected in the IHAC cohort, but also by the presence of HIV positions previously identified as HLA associated, and with different HLA specificities and/or target amino acids in the two cohorts. The extent to which these unique HLA-associated footprints represent a real biological phenomenon and not a statistical effect will have to be further assessed with experimental data; nevertheless, evidence presented in this study strongly suggests the existence of real differences between the two cohorts. Although the Mexican cohort was much smaller than the IHAC cohort (the power to detect associations increases dramatically with sample size [14]), resulting in only 20% of the expected associations being confirmed in the present cohort, the fact that 53% of the HLA-HIV codon associations were novel in the Mexican cohort strongly suggests differences in HLA-mediated evolution between the two clade B-infected cohorts. Although these novel associations may represent false negatives from the IHAC cohort, that cohort is large enough that the false negative rate is expected to be quite small and any false negatives are likely to be rare events in it [14]. It is also possible that the novel associations represent false positives in the present cohort; however, with an expected 20% false-positive rate due to the q < 0.2 threshold, the number of novel associations found in the Mexican cohort is striking. In addition, of the 18 novel HLA-HIV codon pairs in the Mexican cohort, at least 11 (61%) can be explained by confirmed or potential CTL epitopes (Figure 6), strongly arguing for the validity of these associations and for the existence of real biological differences in HLA-mediated selection between the two cohorts

The observation of point differences in the population consensus sequences of the two cohorts which were mapped to HLA-associated sites is a piece of evidence that further supports the differential impact of HLA selection in HIV evolution at the population level (Table 3). This was the case of position RT 277, associated with A*03 both in the Mexican cohort and in the IHAC cohort, in which the adapted form 277R has become fixed in the IHAC consensus while the susceptible form 277K has remained in the Mexican consensus. Not surprisingly, the frequency of A*03 was three times higher in the HOMER cohort than in the Mexican cohort (p = 7.08E-10, q = 1.00E-08), supporting an important role of HLA allele frequency in the fixation of HLA escape mutations at the population level (Figure 4, Table 3). Similarly, PR 93 was associated with B*15 in the IHAC cohort with the susceptible form 93I observed in this cohort's consensus, but the adapted form 93L observed in the Mexican consensus. Although no direct HLA association was detected in the Mexican cohort at this site (probably due to statistical power issues), position PR 93 was associated with other HIV sites, such as PR 71, which is HLA associated (Figure 6). Thus, changes in population consensus sequences may be linked with HLA-mediated selection. Also of interest is the observation that B*44 was associated with 5 of the 30 HLA-HIV codon pairs identified in the Mexican cohort. Two of these associations have been described in the IHAC cohort and two have been previously identified as HLA-associated positions with different HLA specificities (Figure 6). The strong influence of B*44 on HLA-mediated HIV evolution in the Mexican cohort could reflect differences in immunodominance hierarchies of CTL responses in the context of different HLA frequency distributions. Whereas strongly immunodominant CTL responses could be masking the effect of other less immunodominant responses in one cohort, these responses could have a greater impact on HLA-mediated HIV evolution in another cohort in which the immunodominant responses are infrequent. It is notable that the frequencies of many strongly immunodominant HLA alleles, such as B*57, B*27, B*08, B*07, A*03, and A*11 [25], are lower in the Mexican cohort compared to the IHAC cohort (q < 0.05) (Figure 4). It is possible that in the latter cohort, CTL responses restricted by these alleles could be masking the effect of other less immunogenic alleles that are frequently seen in the Mexican population. Indeed, this could be the case for Cw*07, the most frequent HLA-C allele group in the Mexican cohort, which explains 10% of HLA-HIV codon pairs observed in our analysis. These associations are unique to the Mexican cohort, and are supported by predicted epitopes and/or a strong statistical association (q < 0.05) (Figure 6).

The case of B*39 is also noteworthy, being the most frequent HLA-B allele group in the Mexican cohort with a frequency 7 times higher than that observed in the IHAC cohort (p = 1.80E-44, q = 1.21E-42) (Figure 4). B*39 explained another 10% of the HLA-HIV codon pairs identified in the Mexican cohort, suggesting either a strong influence of this allele in HIV evolution in the immunogenetic context of the Mexican cohort or a higher statistical power to detect associations. Interestingly, B*39-restricted associations were primarily escape associations (where possession of B*39 made it less likely to have the target amino acid in question), in which the target amino acid was a residue other than the consensus, suggesting that the consensus residue represents a possible escaped form for B*39 at this position (Additional file 1: Table S3, Figure 6). This could be suggestive of a frequent role of the B*39 allelic group in HIV codon conservation in the Mexican cohort. This HLA-associated conservation of sites has been previously described with highly frequent HLA alleles that promote the accumulation of CTL adapted variants in different populations [22,23].

Overall, two general key aspects could explain the observation of different associations in cohorts that are infected by viruses of the same clade but which have different HLA frequencies: 1) Different patterns of immunodominance, which argue for real differences in CTL epitope targeting; and 2) Different statistical power to detect associations, which argues for a statistical effect rather than a biological difference. For example, the absence of strong immunodominance patterns in certain populations could potentially facilitate the detection of HIV polymorphisms associated with less immunodominant alleles. Being able to confirm this possibility at the population level strongly relies on low false positive/negative rates. Although the false negative rate on the IHAC data is low, further experimental data is necessary to confirm this point. On the other hand, different HLA frequencies can simply change the statistical power to detect associations, thus supporting the importance of assessing HLA-mediated selection in a diverse set of cohorts. The possibility also exists that a simple statistical power issue could be resolved by combining different cohorts infected by the same viral clade to make a larger reference set, supporting the creation of a universal set of associations that could get updated periodically as new sequences are added. Such a sequence and association database would allow extrapolation from a large reference set to new demographic groups for which collection of cohorts would be difficult. The fact that 17 of the 23 novel HLA-HIV codon associations in the Mexican cohort involved HLA alleles whose frequencies were not significantly different from those in the IHAC cohort strongly suggests that their presence is not due to increased statistical power but rather may be due to differences in patterns of epitope targeting. Furthermore, immunodominance effects as well as statistical power issues depending on HLA frequencies could both exist in the same dataset. Examples of both phenomena have been described above for the Mexican cohort, suggesting that a set of immunogenetically diverse cohorts could greatly enrich HIV evolutionary studies without the need of very large cohorts. It should also be noted that a broad two-digit HLA allele grouping does not reveal all possible divergence in HLA pressure, as a number of HLA subtypes with different peptide-binding motifs can be defined at four-digit level within some allelic groups such as B*35, B*40, B*51, B*58, A*02, all with highly characteristic distributions in different populations [9,49]. Thus, significant divergence in selection in some cases could be explained by different dominant four-digit subtypes of the broad allele group in the compared cohorts. This fact could have an impact on statistical power to detect associations defined by different subtypes within a broad allele group in different populations and further argues for the unique HLA-associated imprinting of HIV in different populations.

In summary, although important limitations exist for the analysis of HLA-mediated HIV evolution in the Mexican population, including the presence of false positive associations and the low power to detect associations, our analysis yielded strong evidence suggesting that unique characteristics in HLA-mediated HIV evolution in the Mexican cohort indeed exist. These include the striking proportion of unique HLA-HIV codon associations in the Mexican cohort (many of which can be supported by predicted or confirmed CTL epitopes), the presence of HLA-associated differences in the consensus sequence with respect to the HOMER consensus (which reflects differential fixation of CTL escape mutations at the population level with a high dependency on HLA frequency), and the existence of a high proportion of novel associations that involve HLA alleles whose frequencies were similar in the Mexican and the IHAC cohorts (which argues against a statistical power issue in detecting at least some of the significant associations).

To further characterize HLA-mediated HIV evolution, HLA-HIV codon and HIV codon-HIV codon associations were compared in free plasma virus and PBMC proviral DNA in the cohort of Mexican individuals. As shown by graphically depicting the PDNs for the two viral compartments, different mutational patterns and different HLA-HIV codon associations were seen in actively replicating plasma viruses and PBMC-archived proviruses at the population level. A significantly lower number of HLA-HIV codon associations was observed in proviral sequences and there were more distinct than shared HIV codon-HIV codon associations in the two compartments (Figure 7). This could be explained by the observation that proviral sequences frequently represent a stable reservoir of HIV sequences archived early in the course of the infection [53], whereas plasma viruses represent sequences from later in the course of infection. Thus, the proviral sequences may have been archived before some epitopes were targeted by host CTL responses, or before escape mutations had a chance of being selected at epitopes already being targeted by CTLs, resulting in fewer associations in proviral sequences than in the extant plasma sequences. Indeed, previous studies have shown the presence of HLA-associated escape mutations in plasma viruses that are rare in proviruses within infected individuals [54]. Nevertheless, proviral HLA-HIV codon pairs could not be mapped to known epitopes of early escape [25] in the present data, although the possibility exists that a larger cohort and analyses in other viral genes could further support this correlation. However, given the differences in escape association that we have observed between the Mexican and IHAC cohorts and the observation that the cohort described [25] is immunogenetically similar to the IHAC cohort, it may be that the discordance between proviral escape associations reported here and previously reported early-escape epitopes reflects different patterns of CTL epitope targeting and kinetics between the two populations. The proviral associations in the Mexican cohort could thus represent early escape events in a Latin American cohort setting.

Surprisingly, some HLA associations detected for proviral sequences were not seen in the plasma virus dataset. Some of these HLA-HIV codon pairs observed exclusively in proviral sequences have fairly high q-values, possibly suggesting the presence of false positive associations. However, unique proviral associations could also suggest a chronological reshaping of HLA-mediated HIV evolution, reflecting rapidly reverting mutations which are lost soon after transmission to HLA-mismatched individuals. Alternatively, the existence of organ compartmentalization of HIV variants within an infected host and its relation to positive selection has been described [55]. This phenomenon could explain population differences between actively replicating viruses coming from a specific compartment with characteristic selective pressures and archived proviruses, remaining as reservoir(s) originating from different anatomical and/or cellular compartments.

Shared associations between the plasma virus and the PBMC provirus compartments may reflect sites in the viral proteome with continuous CTL targeting throughout the chronic infection, a characteristic that might be of interest in the selection of candidate vaccine targets. On the other hand, these apparently more stable associations could also reflect epitopes with early CTL targeting that has stopped, but for which no reversion has occurred, suggesting low fitness costs for escape. If the latter case were true, some shared associations might be more likely to reach fixation at the population level in the future. This would have implications for our understanding and predictive capabilities of HIV adaptation in human populations.

Similarly, unique coevolving HIV codon pairs were detected in proviral sequences and in plasma virus sequences, perhaps reflecting different patterns of compensatory mutations to the different HLA escape mutations observed in the two compartments. Alternatively, unique proviral HIV codon-HIV codon pairs could be explained as a reorganization of mutational patterns in HIV evolution that reflect escape mutations selected in previous hosts as well as new mutations selected in the current host, while unique plasma virus HIV codon-HIV codon pairs could reflect sequential footprints left by viral adaptation to HLA-restricted responses in chronic infection in the current host. These observations bring up interesting consequences for our understanding of HLA-mediated HIV evolution, suggesting that the appearance and density of the PDNs for a specific population are highly dynamic and could vary in time. The dynamic development of CTL responses over the course of infection within an individual has been previously reported [51,52]. Further studies in follow-up cohorts or in carefully stratified cross-sectional cohorts might be able to support or refute these observations.

## Conclusion

In conclusion, our data derived from analysis of HLA-mediated HIV evolution in a previously uncharacterized, immunogenetically unique cohort from Central/Southern Mexico, support a highly conserved and strongly predictable component of HLA-mediated HIV evolution at the population level, resulting in HLA-associated footprints in the circulating virus worldwide. This effect is evident even after considering important objections to the HLA-HIV population imprinting hypothesis, such as the rapid reversion of a considerable part of the total CTL escape mutations in the absence of the selective HLA allele [56], the complexity of the CTL response which frequently imposes conflicting selective forces in the same site of the viral sequence [27], the possibility that an escape variant selected by a specific HLA allele can be targeted by CTL responses restricted by different HLA alleles [18,30,57], and the dynamic immunodominance hierarchies observed in HLA-restricted responses [51,52]. Interestingly, the HLA-mediated evolution analysis in our cohort of Mexican individuals showed additional HLA-HIV codon associations that have not been described in previously studied cohorts, including the large multi-center IHAC cohort with a clearly different immunogenetic background to the Mexican cohort. This fact supports the possibility that these specific associations are not significantly impacting HIV evolution at the population level in other cohorts, but that they are significant in the immunogenetic context of the Mexican population. Comparative HLA-mediated HIV evolution studies, with comparable methods that take into account important confounding factors such as HIV codon covariation, HIV lineage effects and HLA linkage disequilibrium, can thus be useful in identifying these distinct HLA-associated footprints in different populations worldwide. Extending such comparative studies to other immunogenetically distinct cohorts, would allow the reconstruction of a more complete panorama of the impact of HLA selection in HIV evolution worldwide. This knowledge may prove useful for the development of vaccine candidates and the development of therapeutic strategies directed to specific populations. The creation of a universal database of HLA-associated HIV sites applying comprehensive and comparable models to assess HLA-mediated evolution in immunogenetically divergent cohorts from different parts of the world, including cohorts predominantly infected by different viral clades, would greatly improve our understanding of HIV evolution worldwide. Importantly, further experimental evidence will help to understand the limitations imposed by statistical models to detect footprints of HLA-associated evolution in HIV in different populations.

Additionally, a comparison between HLA-mediated evolution in free plasma virus and PBMC proviral sequences suggested a highly dynamic HLA-associated evolution in HIV, as many of the HLA-HIV codon associations observed in the free plasma virus compartment are not evident in the proviral dataset, which is presumably enriched in early HIV sequences and does not reflect the full extent of within-host HLA-driven viral evolution. Moreover, shared HLA-HIV codon associations in both viral compartments could be of interest, reflecting epitopes with continuous CTL targeting throughout the chronic infection or, alternatively, escape mutations with low fitness costs that could reach fixation at the population level in the future. Further studies with larger cohorts and various viral genes could enrich these primary observations and increase our understanding of HIV adaptation to different populations worldwide.

## Competing interests

The authors declare that they have no competing interests.

## Authors' contributions

SAR, CEO, EE and GRT conceived and directed the project. SAR wrote the manuscript. CEO carried out and revised statistical analyses. JMC and DH carried out HLA-mediated HIV evolution analyses applying the PDN model. SAR, HVP, and JBH carried out HLA typing. SAR and JBH carried out proviral HIV *pol *sequencing. DGR and CGM carried out HIV *pol *sequencing and coordinated shipping and processing of blood samples. ZLB allowed the use of the HOMER cohort and provided data for the comparisons with the Mexican cohort. SM and MJ allowed the use of the Perth and the USA cohort data. SAR, EE, GRT, CEO, JMC, DH, ZLB, MJ and SM were involved in critically revising the manuscript.

## Supplementary Material

Additional file 1 **Two additional figures and three additional tables**. Figure S1 – Allelic and population frequencies for class I HLA-A, B and C genes in a cohort of HIV-infected individuals from Central/Southern Mexico. Frequencies were obtained with the HLA Frequency Analysis Tool of the Los Alamos HIV Immunology Database . HLA typing was carried out by SSP-PCR as described in the Methods. A total of 292 individuals (584 HLA alleles) were included; 19, 29 and 14 distinct allelic groups were observed for the HLA-A, B and C genes respectively. Figure S2 – Phylogeny of 280 HIV *pol *sequences from a cohort of antiretroviral treatment-naïve individuals from Central/Southern Mexico. A Neighbor-Joining tree was inferred through the analysis of 1305 bp *pol *sequences including the whole protease and 335 codons of the RT in MEGA 4.0. The consensus tree from 1000 bootstrap replicas is shown. Evolutionary distances were calculated with Kimura's two-parameter model and are shown in substitutions per site. Positions with missing information were eliminated by pairwise comparison. 93 reference sequences of the main HIV-1 groups, subtypes and recombinant forms, obtained from the Los Alamos HIV Sequence Database  were included. Reference subtype B sequences are shown in blue, reference sequences of all other subtypes and recombinant forms are shown in red and Mexican sequences are shown in black. The inset shows a detail of the Mexican sequence cluster. Table S1 – HLA-A, B and C population and allelic frequencies in the Mexican cohort. Table S2 – Linkage disequilibrium for class I HLA-A, B and C genes in the Mexican cohort. Table S3 – Protease and RT HLA-HIV codon associations observed in the Mexican cohort. Click here for file
