# Supplementary material for: Unique features of HLA-mediated HIV evolution in a Mexican cohort: a comparative study
Source: Retrovirology. 2009 Aug 10;6:72. doi: 10.1186/1742-4690-6-72 (PMC2734549; doi:10.1186/1742-4690-6-72)
Supplement: Additional file 1 — Two additional figures and three additional tables. Figure S1 – Allelic and population frequencies for class I HLA-A, B and C genes in a cohort of HIV-infected individuals from Central/Southern Mexico. Frequencies were obtained with the HLA Frequency Analysis Tool of the Los Alamos HIV Immunology Database . HLA typing was carried out by SSP-PCR as described in the Methods. A total of 292 individuals (584 HLA alleles) were included; 19, 29 and 14 distinct allelic groups were observed for the HLA-A, B and C genes respectively. Figure S2 – Phylogeny of 280 HIV pol sequences from a cohort of antiretroviral treatment-naïve individuals from Central/Southern Mexico. A Neighbor-Joining tree was inferred through the analysis of 1305 bp pol sequences including the whole protease and 335 codons of the RT in MEGA 4.0. The consensus tree from 1000 bootstrap replicas is shown. Evolutionary distances were calculated with Kimura's two-parameter model and are shown in substitutions per site. Positions with missing information were eliminated by pairwise comparison. 93 reference sequences of the main HIV-1 groups, subtypes and recombinant forms, obtained from the Los Alamos HIV Sequence Database were included. Reference subtype B sequences are shown in blue, reference sequences of all other subtypes and recombinant forms are shown in red and Mexican sequences are shown in black. The inset shows a detail of the Mexican sequence cluster. Table S1 – HLA-A, B and C population and allelic frequencies in the Mexican cohort. Table S2 – Linkage disequilibrium for class I HLA-A, B and C genes in the Mexican cohort. Table S3 – Protease and RT HLA-HIV codon associations observed in the Mexican cohort. [file 1742-4690-6-72-S1.pdf]

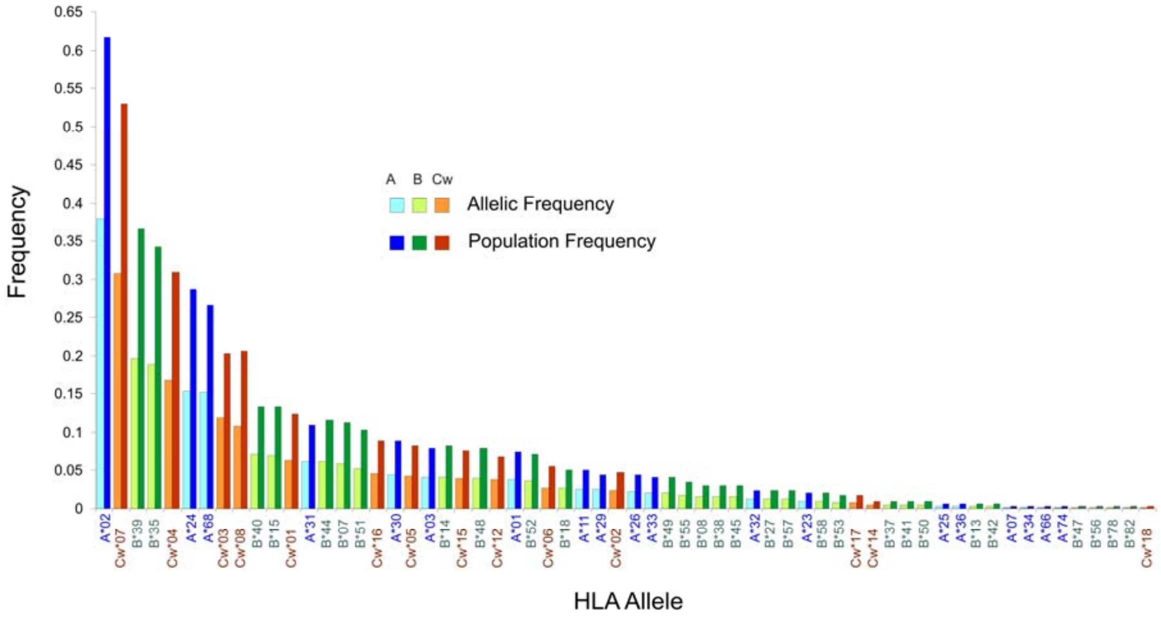

Figure S1 - Allelic and population frequencies of class I HLA-A, B and C genes in a cohort of HIV-infected individuals from Central/Southern Mexico.

Frequencies were obtained with the HLA Frequency Analysis Tool of the Los Alamos HIV Immunology Database (www.hiv.lanl.gov/content/immunology/tools-links.html). HLA typing was carried out by SSP-PCR as described in the Methods. A total of 292 individuals (584 HLA alleles) were included; 19, 29 and 14 distinct allelic groups were observed for the HLA-A, B and C genes respectively.

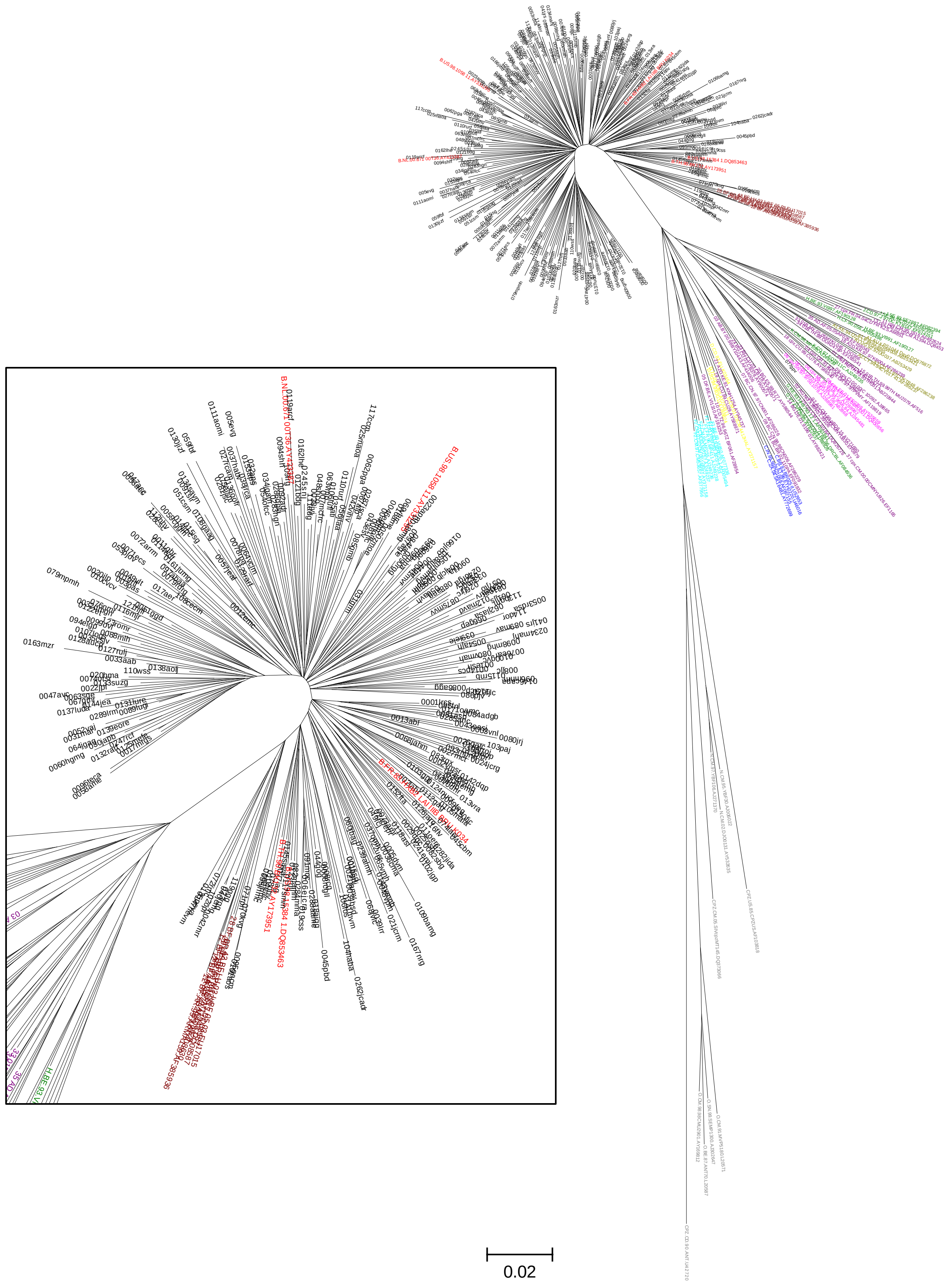

Figure S2 - Phylogeny of 280 HIV *pol* sequences from a cohort of antiretroviral treatment-naïve individuals from Central/Southern Mexico.

A Neighbor-Joining tree was inferred through the analysis of 1305 bp *pol* sequences including the whole protease and 335 codons of the RT in MEGA 4.0. The consensus tree from 1000 bootstrap replicas is shown. Evolutionary distances were calculated with Kimura's two-parameter model and are shown in substitutions per site. Positions with missing information were eliminated by pairwise comparison. 93 reference sequences of the main HIV-1 groups, subtypes and recombinant forms were obtained from the Los Alamos HIV Sequence Database (http://www.hiv.lanl.gov/content/sequence/NEWALIGN/align.html) and included in the tree. Black: Mexican sequences, red: subtype B, dark red: BF recombinant forms, olive: subtype A, dark blue: subtype C, yellow: subtype D, light blue: subtype F, green: subtypes G, H, J, K, fucsia: 06_cpx recombinant forms, purple: other recombinant forms, grey: O-N groups and SIVs. The inset shows a detail of the Mexican sequence cluster.

Table S1 – HLA-A, B and C population and allelic frequencies in the Mexican cohort. §

HLA	Allelic Frequency	Population Frequency
A*02	0.3784	0.6164
Cw*07	0.3076	0.5292
B*39	0.1969	0.3664
B*35	0.1884	0.3425
Cw*04	0.1684	0.3093
A*24	0.1541	0.2877
A*68	0.1524	0.2671
Cw*03	0.1186	0.2027
Cw*08	0.1082	0.2062
B*40	0.0719	0.1336
B*15	0.0702	0.1336
Cw*01	0.0636	0.1237
A*31	0.0616	0.1096
B*44	0.0616	0.1164
B*07	0.0582	0.113
B*51	0.0531	0.1027
Cw*16	0.0464	0.0893
A*30	0.0445	0.089
Cw*05	0.043	0.0825
A*03	0.0411	0.0788
B*14	0.0411	0.0822
Cw*15	0.0395	0.0756
B*48	0.0394	0.0788
Cw*12	0.0378	0.0687
A*01	0.0377	0.0753
B*52	0.036	0.0719
Cw*06	0.0275	0.055
B*18	0.0274	0.0514
A*11	0.0257	0.0514
A*29	0.0257	0.0445
Cw*02	0.0241	0.0481
A*26	0.0223	0.0445
A*33	0.0205	0.0411
B*49	0.0205	0.0411
B*55	0.0171	0.0342
B*08	0.0154	0.0308
B*38	0.0154	0.0308
B*45	0.0154	0.0308
A*32	0.012	0.024
B*27	0.012	0.024
B*57	0.012	0.024
A*23	0.0103	0.0205

B*58	0.0103	0.0205
B*53	0.0086	0.0171
Cw*17	0.0086	0.0172
Cw*14	0.0052	0.0103
B*37	0.0051	0.0103
B*41	0.0051	0.0103
B*50	0.0051	0.0103
A*25	0.0034	0.0068
A*36	0.0034	0.0068
B*13	0.0034	0.0068
B*42	0.0034	0.0068
A*07	0.0017	0.0034
A*34	0.0017	0.0034
A*66	0.0017	0.0034
A*74	0.0017	0.0034
B*47	0.0017	0.0034
B*56	0.0017	0.0034
B*78	0.0017	0.0034
B*82	0.0017	0.0034
Cw*18	0.0017	0.0034

§ HLA typing was carried out by SSP-PCR as described in Methods. A total of 292 individuals and 584 alleles were included.

Table S2 - Linkage disequilibrium for class I HLA-A, B and C genes in the Mexican cohort. §

HLA1	HLA2	p-Value	Both	Only HLA1	Only HLA2	None	P(HLA1)	P(HLA2)	P(HLA1 & HLA2)	E(P(HLA1 & HLA2))
B*35	Cw*04	1.30E-33	78	22	11	177	0.3472	0.3090	0.2708	0.1073
B*39	Cw*07	4.40E-21	97	10	55	126	0.3715	0.5278	0.3368	0.1961
B*15	Cw*01	8.99E-15	25	13	10	240	0.1319	0.1215	0.0868	0.0160
B*40	Cw*03	1.08E-11	29	10	29	220	0.1354	0.2014	0.1007	0.0273
B*14	Cw*08	5.83E-11	22	2	38	226	0.0833	0.2083	0.0764	0.0174
B*48	Cw*08	3.63E-10	21	2	39	226	0.0799	0.2083	0.0729	0.0166
B*44	Cw*16	3.58E-07	16	18	9	245	0.1181	0.0868	0.0556	0.0102
B*40	Cw*07	2.84E-06	3	36	149	100	0.1354	0.5278	0.0104	0.0715
B*38	Cw*12	1.02E-05	8	1	12	267	0.0312	0.0694	0.0278	0.0022
B*44	Cw*05	2.66E-05	14	20	9	245	0.1181	0.0799	0.0486	0.0094
B*18	Cw*05	3.92E-04	9	5	14	260	0.0486	0.0799	0.0312	0.0039
A*03	B*07	4.23E-04	12	9	19	248	0.0729	0.1076	0.0417	0.0078
A*01	B*57	7.22E-04	6	15	0	267	0.0729	0.0208	0.0208	0.0015
B*41	Cw*17	2.54E-02	3	0	2	283	0.0104	0.0174	0.0104	0.0002
B*07	Cw*07	4.64E-02	28	3	124	133	0.1076	0.5278	0.0972	0.0568
A*30	B*39	5.35E-02	0	25	107	156	0.0868	0.3715	0.0000	0.0323
B*52	Cw*03	6.68E-02	13	7	45	223	0.0694	0.2014	0.0451	0.0140
A*33	B*14	6.88E-02	7	5	17	259	0.0417	0.0833	0.0243	0.0035
B*51	Cw*15	9.22E-02	10	20	12	246	0.1042	0.0764	0.0347	0.0080
A*29	Cw*16	1.92E-01	7	6	18	257	0.0451	0.0868	0.0243	0.0039
A*30	B*18	3.63E-01	7	18	7	256	0.0868	0.0486	0.0243	0.0042

§ 288 individuals from Central/Southern Mexico were included. Both: number of individuals expressing HLA1 and HLA2. Only HLA1: number of individuals expressing HLA1, but not HLA2. Only HLA2: number of individuals expressing HLA2, but not HLA1. None: number of individuals expressing neither HLA1 nor HLA2. P(HLA1): observed frequency of individuals expressing HLA1 in the cohort. P(HLA2): observed frequency of individuals expressing HLA2 in the cohort. P(HLA1 & HLA2): observed frequency of individuals expressing both HLA1 and HLA2 in the cohort. E(P(HLA1 & HLA2)): expected frequency of P(HLA1 & HLA2) if P(HLA1) and P(HLA2) were independent. The p-value for two-tail Fisher exact tests is shown. Estimation of the probability that P(HLA1) and P(HLA2) are independent given the observed information. A low p-value indicates that the occurrence of HLA1 and HLA2 is linked in the cohort. Number of two-way tests performed = A*B+A*C+B*C = 1190; p-value for two-way comparisons = 0.05/(A*B+A*C+B*C) = 4.202e-05 for a confidence level of 95%.

Table S3 - Protease and RT HLA-HIV codon associations observed in the Mexican cohort. §

Predictor Variable (HLA)	Target Variable (HIV codon)	TT	TF	FT	FF	p-value	q-value	Conditioning Variables (Coevolving codons, conditioning HLA)	Reference	Observations
A*03 [b]	RT 277R	19	2	56	195	1.20E-10	1.82E-06		[34], [25], [37]	
A*03 [a]	RT 277K	2	19	195	56	1.20E-10	1.82E-06		[34], [25], [37]	
A*30 [a]	RT 281K	18	5	251	3	0.00073383	0.13153195		[37]	
A*33 [a]	RT 122P	1	11	67	191	0.0009406	0.14940166	RT 98A [c]	[37]†	Consistent (Escape from P in Mex, attraction to E in IHAC).
B*07 [b]	RT 165I	7	18	10	241	0.0005105	0.1175217		[34], [37]	
B*07 [a]	RT 165T	18	7	240	10	0.00052215	0.1175217		[34], [37]	
B*08 [a]	RT 272A	1	7	122	143	0.00174628	0.19533002		[25]†, [37]†	Different (B*42 escape from P in IHAC, B*08 has a much lower frequency in Mex).
B*18 [b]	RT 138A	3	10	1	266	0.00013697	0.05164684		[25], [37]	
B*18 [a]	RT 138E	10	3	265	1	0.00013862	0.05164684		[25], [37]	
B*18 [b]	RT 245E	4	8	22	240	0.00144187	0.18595855	PR 45K [c], B58 [b]	[34]†, [37]†	Different (B*57/58 escape from V to L, B*44 escape from E in IHAC; no B*18 association with p<0.05 in IHAC, no difference in HLA frequency).
B*27 [a]	RT 135I	1	6	153	94	0.00140863	0.1832374	B*51 [a], RT 202I [d], PR 12N [a]	[34]†, [25]†, [37]†	Different (B*51/52 escape from I to T/M in IHAC; B*27 less frequent in Mex).
B*39 [a]	PR 71V	6	85	23	140	0.00121556	0.17636733	PR 93L [d], PR 57K [b]	[25]†, [37]†	Different (B*53 escape from T in IHAC; B*39 much more frequent in Mex).
B*39 [a]	RT 102R	0	96	17	153	0.00011626	0.04678005	B*48 [b], RT 64K [a]	[34]†, [37]†	Different (B*48 K to R; B*39 much more frequent in Mex).
B*39 [b]	RT 64R	5	91	4	166	0.00166664	0.1905207	RT 102R [d]	NR	No close association in IHAC.
B*39 [a]	RT 64K	91	5	166	4	0.00166664	0.1905207	RT 102R [c]	NR	No close association in IHAC.
B*40 [b]	RT 202V	5	27	10	186	0.00100069	0.15487079	RT 135I [a], RT 197E [b], PR 35E [d], RT 334L [a], RT 165I [d], RT 122P [b]	NR	No close association in IHAC; no difference in HLA frequency among cohorts.
B*40 [a]	RT 202I	27	5	186	10	0.00100069	0.15487079	RT 135I [b], RT 197E [a], PR 35E [c], RT 334L [b], RT 165I [c], RT 122P [a]	NR	No close association in IHAC; no difference in HLA frequency among cohorts.
B*44 [b]	PR 35D	21	10	33	210	2.78E-09	1.40E-05		[34], [25], [37]	
B*44 [a]	PR 35E	9	22	210	33	6.65E-10	6.69E-06		[34], [25], [37]	
B*44 [b]	RT 211K	22	6	106	115	0.00185231	0.19533002	PR 43K [c], RT 196E [a]	[34]†, [37]†	Consistent (R to K in Mex, just escape from R in IHAC).
B*44 [a]	RT 211R	2	28	94	128	4.19E-05	0.02767678		[34], [37]	

B*44 [a]	RT	329I	14	15	203	30	0.00130602	0.18323349	RT 334L [b]	[37]†	Different (B*53 escape from V in IHAC; B*44 less frequent in Mex).
B*44 [d]	RT	123N	7	25	3	236	0.00152115	0.18684048	RT 122E [d]	[34]†, [25]†	Different (B*35 D to E in IHAC).
B*44 [c]	RT	68S	26	7	232	7	0.00185709	0.19533002	PR 41R [c], RT 139T [d]	NR	No close association in IHAC.
B*44 [d]	RT	68G	7	26	7	232	0.00185709	0.19533002	PR 41R [d], RT 139T [c]	NR	No close association in IHAC.
B*45 [b]	RT	200A	5	3	37	220	0.00072663	0.13153195		[34]†, [37]†	Different (B*40/41 attraction to I, B*08 escape from A, B*37 escape from T in IHAC; no difference in B*45 frequency).
B*48 [b]	RT	102R	10	8	7	249	2.54E-09	1.40E-05		[34], [37]	
B*48 [a]	RT	102K	8	10	227	28	2.83E-05	0.02083046		[34], [37]	
B*49 [b]	RT	50V	3	9	3	265	0.00078914	0.13651316		NR	No close association in IHAC; no difference in HLA frequency among cohorts.
B*49 [a]	RT	50I	9	3	265	3	0.00078914	0.13651316		NR	No close association in IHAC; no difference in HLA frequency among cohorts.
B*51 [b]	PR	12S	5	23	2	249	7.70E-05	0.03639581		[34]†, [37]†	Consistent (escape from T, weak attraction to S [p=0.02] in IHAC).
B*51 [b]	PR	14R	10	13	20	212	0.00064885	0.13057972	RT 135I [a]	[34], [37]†	
B*51 [a]	PR	14K	12	10	212	20	0.00052685	0.1175217	RT 135I [b]	[34], [37]†	
B*51 [a]	RT	135I	4	20	155	84	6.58E-08	0.00016543		[34], [25]†, [37]	
B*52 [b]	PR	12A	7	8	9	247	2.44E-06	0.00319527		[34], [37]†	
B*52 [a]	PR	12T	4	13	215	37	9.21E-08	0.00019546		[34], [37]†	
B*57 [b]	RT	297A	4	3	33	239	0.0014485	0.18601806		NR	No close association in IHAC (B*58 has attraction to R with p=0.03, q=0.88).
Cw*04 [a]	RT	248D	0	88	11	180	0.00160017	0.18790507		[25]†, [37]†	Different (Cw*02 escape from E, B*15 escape from D in IHAC; B*15 appears to be in slight negative LD with Cw*04).
Cw*07 [c]	RT	276V	111	32	116	8	7.21E-05	0.03639581		NR	The attraction to V is nearly seen as a Cw*08 association in IHAC (q=0.22); no difference in HLA frequency among cohorts.
Cw*07 [d]	RT	276T	11	131	1	130	0.00152896	0.18684048	RT 272P [d]	NR	The attraction to V is nearly seen as a C*08 association in IHAC (q=0.22); no difference in HLA frequency among cohorts.
Cw*07 [a]	RT	179D	0	147	8	125	0.00061063	0.12536221		NR	No close association in IHAC; Cw*08 is weakly assoc with escape from V (p=0.006, q=0.42).
Cw*07 [c]	RT	278Q	137	8	129	1	0.00130929	0.18323349	RT 281R [a]	[25]†, [37]†	Different (Cw*12 attraction to V is somewhat close, q=0.22).
Cw*15 [a]	PR	18Q	18	4	253	4	0.00118543	0.17492044		NR	No close associations (p<0.05) in IHAC; no difference in HLA frequency among cohorts.

§ 280 individuals from Central/Southern Mexico were included. Associations with q<0.2 are shown. NR – Not previously reported associations. †HIV codon has been previously reported to be under HLA pressure, but HLA specificity and/or target amino acid are different to that observed in the present study. TT, TF, FT, FF: 2x2 contingency tables; HLA is the first value, amino acid is the second value. [a] escape (having the predictor makes it less likely to have the target), [b] attraction (having the predictor makes it more likely to have the target), [c] reversion (not having the predictor makes it more likely to have the target), [d] repulsion (not having the predictor makes it less likely to have the target).
